# Atomic Force Microscopy beyond Topography: Chemical Sensing of 2D Material Surfaces through Adhesion Measurements

**DOI:** 10.1021/acsami.3c19254

**Published:** 2024-04-03

**Authors:** Isaac Brotons-Alcázar, Jason. S. Terreblanche, Silvia Giménez-Santamarina, Gerliz M. Gutiérrez-Finol, Karl S. Ryder, Alicia Forment-Aliaga, Eugenio Coronado

**Affiliations:** † Instituto de Ciencia Molecular (ICMol), Universitat de València, C/Catedrático José Beltrán Martínez, 2, 46980 Paterna, Spain; ‡ Center for Sustainable Materials Processing, School of Chemistry, University of Leicester, University Road, LE1 7RH Leicester, U.K.

**Keywords:** 2D materials, adhesion, atomic force microscopy, MnPS_3_, molecular functionalization, mechanical properties

## Abstract

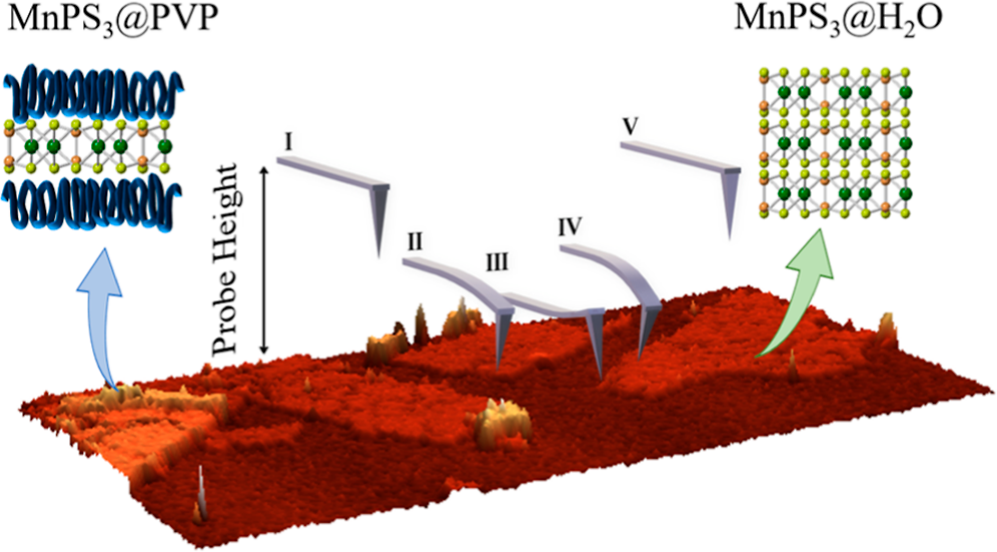

Developing new functionalities of two-dimensional materials (2Dms) can be achieved by their chemical modification with a broad spectrum of molecules. This functionalization is commonly studied by using spectroscopies such as Raman, IR, or XPS, but the detection limit is a common problem. In addition, these methods lack detailed spatial resolution and cannot provide information about the homogeneity of the coating. Atomic force microscopy (AFM), on the other hand, allows the study of 2Dms on the nanoscale with excellent lateral resolution. AFM has been extensively used for topographic analysis; however, it is also a powerful tool for evaluating other properties far beyond topography such as mechanical ones. Therefore, herein, we show how AFM adhesion mapping of transition metal chalcogenide 2Dms (i.e., MnPS_3_ and MoS_2_) permits a close inspection of the surface chemical properties. Moreover, the analysis of adhesion as relative values allows a simple and robust strategy to distinguish between bare and functionalized layers and significantly improves the reproducibility between measurements. Remarkably, it is also confirmed by statistical analysis that adhesion values do not depend on the thickness of the layers, proving that they are related only to the most superficial part of the materials. In addition, we have implemented an unsupervised classification method using k-means clustering, an artificial intelligence-based algorithm, to automatically classify samples based on adhesion values. These results demonstrate the potential of simple adhesion AFM measurements to inspect the chemical nature of 2Dms and may have implications for the broad scientific community working in the field.

## 1 Introduction

Two-dimensional materials (2Dms) are a hot topic of great interest in materials science, both from fundamental and applied points of view.^1^ Nowadays, many different 2Dms are known,^2−4^ which exhibit a variety of physical and chemical properties, including structural,^5^ electronic,^6^ catalytic,^7^ or magnetic,^8^ among others.

The preparation of atomically thin 2Dms employs two different methodologies, namely, bottom-up and top-down approaches. While the first one relies on the synthesis of the ultrathin layers from their atomic or molecular components, the second one hinges on the mechanical or chemical exfoliation of laminar bulk materials down to single layers using chemical, mechanical, dry, or wet methods, which strongly influence the final quality and properties of the resulting 2Dm.^1^

Apart from obtaining new 2Dms, many researchers have focused on modifying the properties of existing ones at will. A possible approach consists of combining 2Dms with 0D, 1D, or 2D nanomaterials, giving rise to new heterostructures with hybrid and synergic properties.^9−11^ Another possibility is chemically functionalizing 2D layers with molecular systems able to protect them or tune their properties.^12−14^

This emergent research field went along with the improvement and development of several surface characterization techniques, pursuing spatially resolved chemical and physical characterization of 2Dms, composites, and heterostructures. These techniques try to evaluate in detail the effect that different treatments have on the surface of all of these materials, which is a key point for their implementation in further applications. Still, a complete evaluation of the intrinsic mechanical and chemical properties of bare or modified 2Dms is difficult to perform.

In this scenario, some studies have been conducted using spectroscopic techniques to analyze 2Dms’ surfaces and determine their chemical composition. One of the most useful techniques used in this case is confocal Raman imaging of 2D materials. This technique is very interesting because it is possible to spatially resolve the chemical composition of materials with good selectivity. However, this method offers a restricted spatial resolution because of the diffraction limit of light and relative sensitivity. Tip-enhanced Raman spectroscopy is another alternative; however, it is a much more complex method and less accessible for most researchers.^15^

Recently, the mechanical properties of several 2D-based heterostructures have been assessed at the nanoscale using either nanoindentators^16^ or atomic force microscopes.^17^ The use of a nanoindentator ensures easier measurements and more reproducible results; however, these techniques produce large trenches on the inspected sample of tens to hundreds of nanometers, hindering the effect of the top layers. As our targets are 2Dms, it is necessary to use a technique that can scan and inspect a surface with nanoscale resolution; hence, atomic force microscopy (AFM) is proposed as an extremely potent tool. Moreover, to further enhance the interaction with the surface and improve its analysis, some authors customize the AFM probes at will.^18^

**Figure 1:**
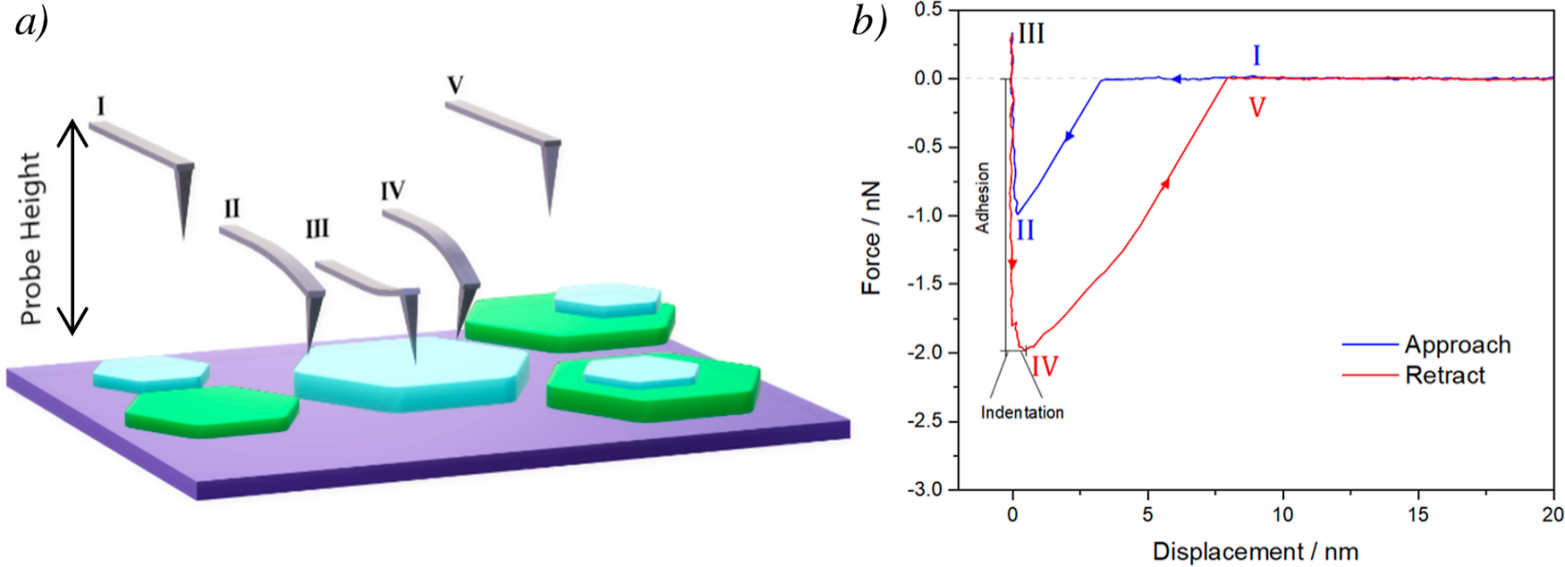
Sketch of the PeakForce tapping mode process in AFM measurements. (a) Steps of a probe cantilever upon approaching a surface: (I) approach, (II) contact, (III) bending, (IV) retract, and (V) free probe. (b) Force–distance curve obtained by an AFM probe on a surface.

The study of mechanical properties by means of an atomic force microscope is based on performing several approach–retract cycles of a probe over a surface, giving rise to force–distance graphs, which are the foundation of the PeakForce tapping mode AFM method used in this work. The different steps of the process are schematically represented in Figure 1.

From the resulting curves, values of different mechanical properties of the material under inspection can be extracted, including adhesion (ability to stick), Young’s modulus (stiffness or elasticity of the material), rupture force, and indentation depth (how much the tip can penetrate the sample at a given load). A more detailed description about the force curves and the method used can be found in the literature^19^ and in Section S1.

From adhesion and indentation data of the force–distance curves, several models can be used to obtain Young’s modulus (*E*) of the sample. However, this is an indirect estimation and requires detailed knowledge about the size and shape of the used probe and a good approximation between theory and real probe-sample interaction.^20^ Note that the size and shape of the probe change along the measurements; hence, a constant characterization of the tip is needed for the correct application of the models. Therefore, the use of properties that can be directly measured with commercially available probes and do not rely on mechanical models of tip–sample interactions is much more convenient. Moreover, avoiding the need for a constant refresh of the probe status clearly simplifies the characterization process.

In this scenario, we show how the use of probe-sample adhesion as the target property to locally inspect a 2Dm represents a more reliable and efficient approach. Nonetheless, it is worth noting that the adhesion force is strongly dependent on two main parameters: the tip–sample contact area and the enviromental conditions (e.g.: temperature or humidity). The former is difficult to know and control as it changes along the measurements.^17^ The latter is a more controllable parameter, but its effect on the measurement is difficult to estimate.^21^ For this reason, it is very difficult to compare data obtained with different probes and/or teams.

In this work, we propose the use of the probe-sample adhesion property to evaluate the behavior of both bare and modified 2Dms with the aim of getting information about the chemical nature of their coating molecules. First, we present a simple data analysis of direct adhesion measurements to extract reliable information on the inspected surfaces. The key strategy proposed to avoid continuous tip-state evaluation and to produce reproducible measurements is to normalize the adhesion data as relative adhesion (RA). The study will be performed with several different AFM probes to find the best one for analyzing the 2Dm’s surface. The analysis of the obtained results is not always evident as it can be difficult to label unequivocally different natural samples manually. Hence, statistical inference methods are used to confirm that samples of different chemical natures can be well classified based on RA values. Analysis of variance (ANOVA) tests are largely used across scientific fields. It is a collection of inexpensive methods with very high accuracy to quantify significant differences between populations of data (which can be applied to large data sets) based on the observed variance of such data.^22,23^ Within ANOVA tests, the Shapiro–Wilk test is a more appropriate method for small sample sizes (<50 samples).^24^ Once the mean values are established for each test and they are significantly different, the next step is to use a proper classifier. One of the most practical methods is called K-means clustering, which can partition samples or variables into clusters based on similarity or the converse.^25^ Having in mind these ideas, we apply this methodology to evaluate the effect that chemical and mechanical exfoliation processes (top-down approach) have on the final surface properties of 2Dms.

As a 2Dm, we have chosen MnPS_3_ due to its chemical stability, ability to host ions or molecules, and the possibility of postfunctionalization after the exfoliation. This is a 2D layered material that belongs to the family of transition metal thiophosphates with the general formula MPX_3_. The materials in this family of compounds exhibit a variety of properties and have applications in fields such as magnetism and catalysis.^4,26^ Moreover, some of them can be covered or functionalized with different molecular materials or capping agents, providing similar topographies with different surface properties. These modified layers will allow us to test their chemical characterization based on their adhesion properties.^13^ In particular, selecting MnPS_3_ with a polyvinylpyrrolidone (PVP) covering is ideal as the polymer seems to get well and homogeneously attached to the 2Dm’s surface, forming a new heterostructure with a well-defined thickness whose roughness is not affected by the molecular capping.^29^ Additionally, we have extended our study to MoS_2_ layers, the well-known layered material extensively used for its potential applications in optoelectronics and electrocatalytic applications,^27,28^ which can also be prepared as bare or PVP-coated layers.^35^

## 2 Results

### 2.1 Sample Description

MPX_3_ is a family of layered materials where M is a transition metal cation (usually Mn, Fe, Co, or Ni) and X is either S or Se. These materials are arranged in stacked 3 atom thick layers where the metallic cations are distributed, forming a honeycomb surrounded by (P_2_S_6_)^2–^ bipyramids.^30^ Several exfoliation methods have been developed for isolating layers of these materials.^31,32^

MnPS_3_-layered material was exfoliated in solution using a previously described chemical approach,^13^ and then, the resulting layers were resuspended in water and in PVP solutions. The obtained samples were MnPS_3_ dispersions called MnPS_3_@H_2_O and MnPS_3_@PVP, respectively. Finally, these dispersions were deposited on Si/SiO_2_ substrates. As the main goal of this work is to discern between both materials through AFM measurements, substrates with both samples mixed on top were also prepared (MnPS_3_@Mix). In addition to these samples, MnPS_3_ crystals were mechanically exfoliated using a mechanical cleavage (“Scotch tape” method) and transferred onto Si/SiO_2_ substrates, forming another sample, MnPS_3_-ME.

Once the MnPS_3_ material was extensively analyzed, some adhesion measurements were done on exfoliated MoS_2_ layers to test the applicability of the method to other materials. MoS_2_ was exfoliated chemically following a method described elsewhere.^33^ On one hand, the obtained layers were kept in H_2_O for measuring bare MoS_2_ layers (MoS_2_@H_2_O samples). On the other hand, some samples were resuspended in PVP solutions (MoS_2_@PVP samples).

### 2.2 Experimental Approach

Adhesion and indentation values directly extracted from the force–distance curves can be used in this study. Although these properties are dependent on the probe state over time (like Young’s modulus does), their values are directly obtained from the force curves performed during the sampling, and they do not rely on any model. Therefore, Young’s modulus determination will rely on the probe state and the feasibility of the model applied, which may not be accurate enough if the probe shape changes drastically. Hence, the readout of adhesion and indentation is more consistent over time. Between these two parameters, it is worth saying that indentation is affected by the first 1–3 nm of the sample in terms of depth (Figure 1a); on the other hand, adhesion is more sensitive to the most superficial part of the sample.^34^ For this reason, the adhesion parameter will be used for the chemical sensing of the surface of 2Dms in the present study.

**Figure 2:**
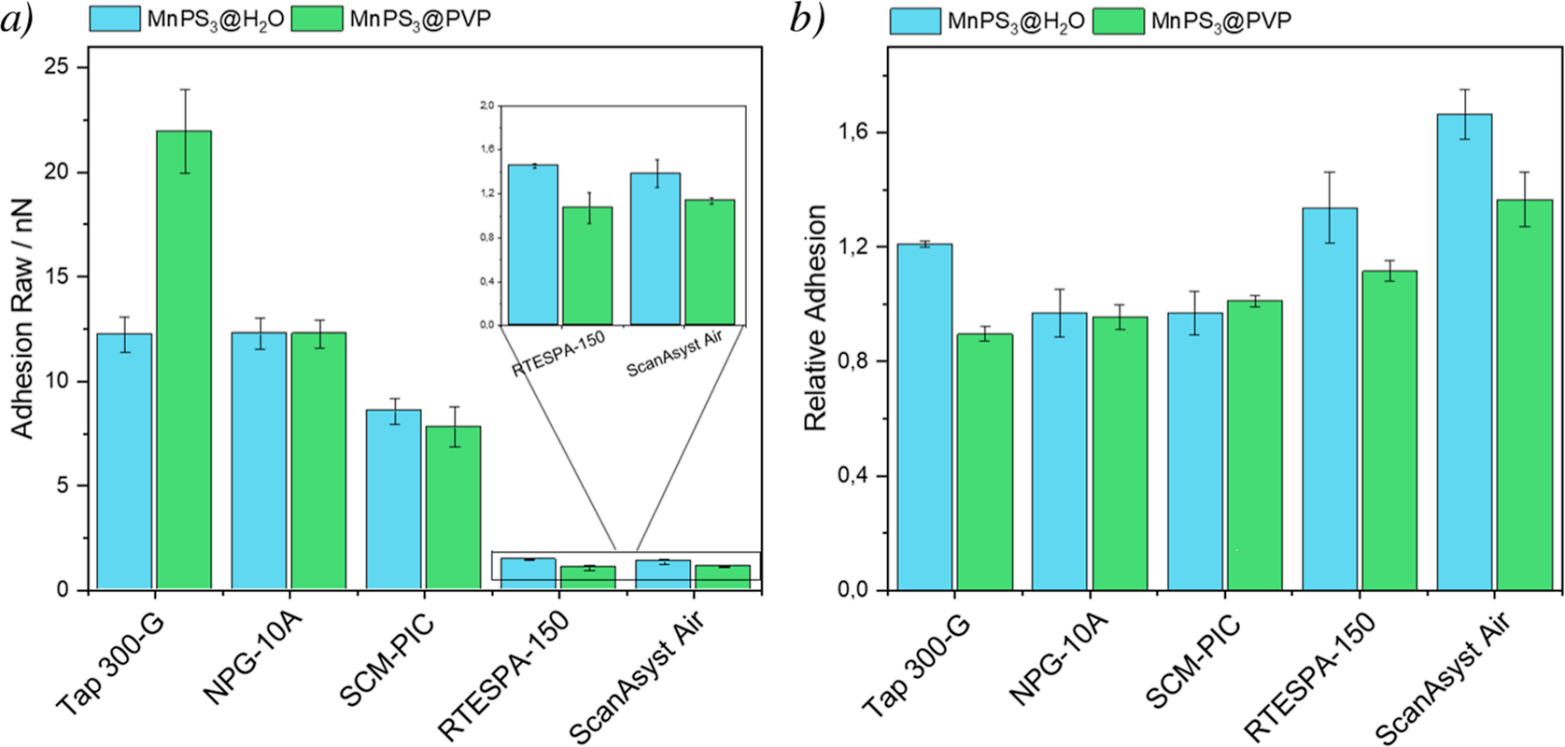
a) Raw adhesion data obtained from measuring MnPS_3_@H_2_O and MnPS_3_@PVP samples with different probes. (b) RA data of the same samples and probes.

Looking for the best sensitivity toward the chemical characterization of the samples, we needed to select the most appropriate probe for our system. Hence, several probes with different parameters or coatings were tested. The selected probes for the study have a wide range of spring constants, from very soft (<1 N/m) to stiff ones (≈40 N/m). Another parameter that can affect the results is, of course, the nature of the probe. Thus, probes with different coatings have also been evaluated (Table S1). MnPS_3_@H_2_O and MnPS_3_@PVP samples were studied with each probe, and the adhesion measured on the flakes was compared for each measurement, Figure 2a.

As was mentioned above, the adhesion is a direct readout from the force–distance curve obtained in the measurement, but it depends on the specific condition of the probe at a given moment plus the force applied for measuring, which may lead to huge variations in the read adhesion values. Nonetheless, the samples we worked with are 2D layers deposited on a Si/SiO_2_ substrate. Therefore, it is possible to simultaneously measure the adhesion on the flakes and on the substrate. This way, we can relate the adhesion measurements of the flakes to the adhesion of the Si/SiO_2_ background and then “normalize” all the values to the Si/SiO_2_. To do so, the mean adhesion values on the 2D layers are divided by the mean adhesion of the background, resulting in RA, or difference between the sample and background, Figure 2b.

For selecting the mean values of adhesion on each area, a combination of topography and adhesion channels was analyzed (see Figures S1–S10). Using the topography data, we can distinguish between 2Dm flakes and the Si/SiO_2_ background through the height threshold. This discrimination between the two surfaces is transferred to the adhesion channel, and thus, adhesion data of flakes and background is obtained for post-treatment.

In Figure 2, we compare raw adhesion results and RA values for five different AFM probes. As can be seen, using raw adhesion makes it impossible to compare results between probes with very different stiffness (i.e., ScanAsyst Air (SAA) vs Tap300-G) as the scales of the values are very different. On the other hand, using RA, all the values are in the same range, making it very easy to compare and select the appropriate probe. A similar comparison has been done using four identical SAA probes (Supporting Information Section 3). The reproducibility and the possibility of comparing results done in different moments or with different probes is one of the highest challenges when analyzing mechanical properties with an atomic force microscope, and this procedure may overcome this limitation.

It is important to consider that when analyzing raw adhesion, even small changes in the size and shape of the probe can make it difficult to compare results. After only one or two scans, the probes may not provide comparable results. However, using RA can help overcome this limitation by ignoring some of the small changes in the probe. This can extend the sensitivity of the probes up to 10 images.

In Figure 2b, we observe the mean RA values obtained with the five different probes under study. From these results, we note that probes with metallic coatings like SCM-PIC and NPG-10A cannot provide good selectivity for the samples. Hence, these probes are discarded for further experiments. A degradation of the coating may be responsible for the poor results of these probes, but some SEM images with elemental analysis have been done after their utilization, and no sign of degradation has been observed (see Figures S11 and S12). Of the last three probes, Tap 300-G and SAA are Si tips, and RTESPA-150 is an antimony-doped Si tip. All these tips allow comparison between MnPS_3_@H_2_O and MnPS_3_@PVP, even though they have different cantilever spring constants and different tip geometries. The spring constants seem to affect the raw adhesion, but the tip material may be the ultimate factor in comparing the RA.

**Figure 3:**
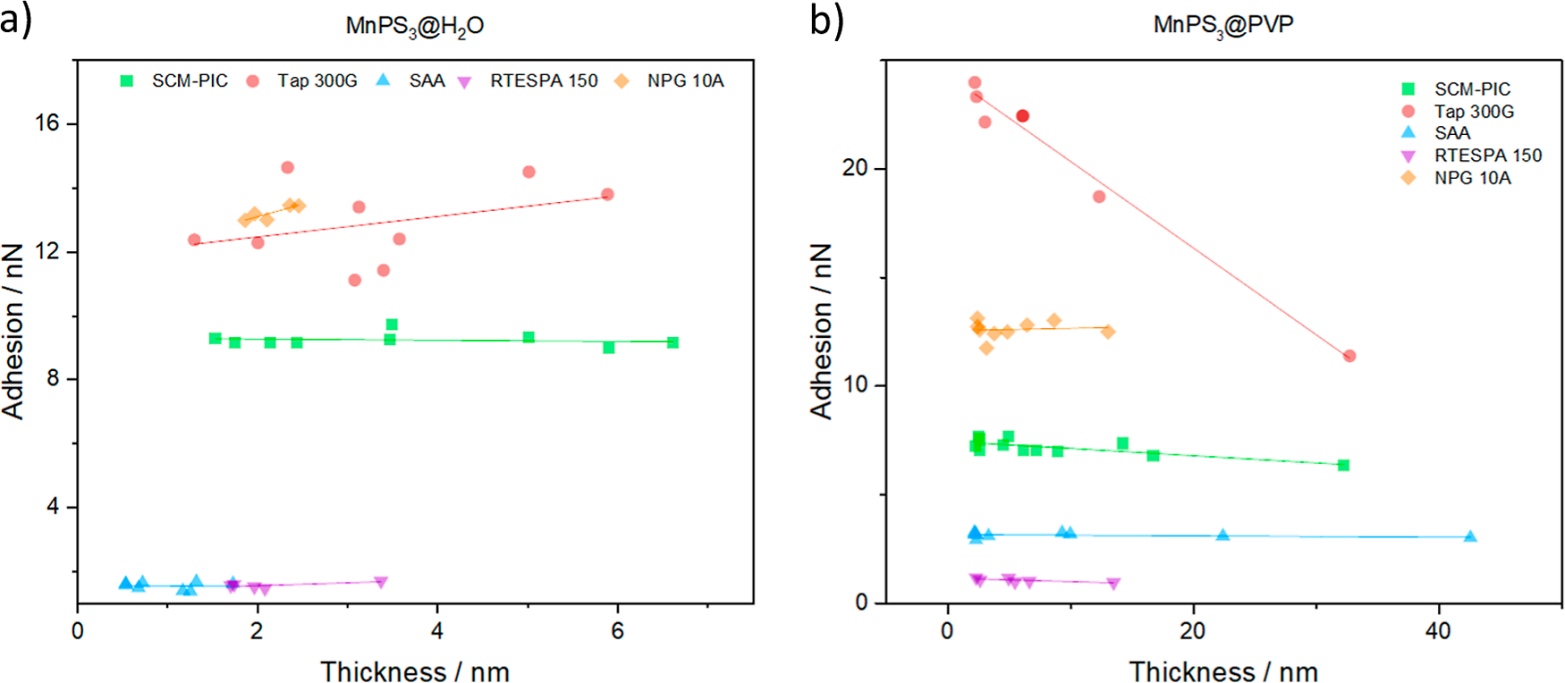
Adhesion–thickness tendency for SCM-PIC, Tap300-G, SAA, and RTESPA probes measured on (a) MnPS_3_@H_2_O samples and (b) MnPS_3_@PVP samples.

Once we have shown that these last three probes are the most promising ones, we have checked that the adhesion signal is not influenced by the thickness of the 2D layers as we are interested in analyzing the signal yielded by the most superficial part of the composite. In Figure 3, the mean thickness and adhesion of each flake in the images used for obtaining adhesion data have been analyzed separately (a detailed analysis of the AFM areas selected can be found in Section S2 and Figures S1c–S10c). Here, we observe how SAA and RTESPA150 probes give almost the same adhesion for all the flakes independently of their thickness, while Tap 300G is clearly affected by the thickness of the material and thus is not an appropriate probe for measuring superficial properties like coatings on 2D layers.

It is worth mentioning that Tap 300-G is a stiffer probe that requires the application of higher probe-surface forces (i.e., PeakForce set point, or PFS) for stabilizing the measurements. However, as can be seen in Section S5, the indentation obtained with Tap 300G is not significantly greater than that obtained with softer probes such as SAA. The effect of PFS on adhesion was also studied for a SAA probe and is discussed in Section S5. In that case, no dependence of the adhesion on the sample thickness was observed even after applying high PFS. Thus, this dependence observed for Tap 300G probes, which are 100 times stiffer than SAA probes, may be related to the difficulty of bending; therefore, an excess of PFS cannot be absorbed by the probe, resulting in excessive adhesion between the tip and the sample. However, so far, all these are preliminary conclusions that should be the subject of further study.

Between the last two probes (SAA and RTESPA-150), both yield a good RA toward both types of samples and do not suffer from any adhesion–thickness dependence. Thus, a deeper analysis of the RA obtained for each sample is conducted with these probes. Additional samples of MnPS_3_@H_2_O and MnPS_3_@PVP are analyzed, and the mean RA value on some selected areas is recorded, as can be seen in Figures S27–S30. An ANOVA test was run on the results, concluding that the RA obtained on MnPS_3_@H_2_O and MnPS_3_@PVP is significantly different; hence, the samples can be distinguished with both types of probes.

### 2.3 Mixed Sample Analysis

After the appropriate probes were selected for testing these samples, the next challenge was to mix both types of flakes on the same substrate and try to identify them. For this purpose, a MnPS_3_@Mix sample was prepared. Here, a Si/SiO_2_ substrate was coated with MnPS_3_@PVP and MnPS_3_@H_2_O together. The objective was to find an area of the substrate where flakes of two different natures are present and to identify them in concordance to the results shown previously.

In a previous study, we suggested that these two types of flakes may be distinguished according to their height (≈1 nm for a monolayer of MnPS_3_@H_2_O and 2.7 nm for monolayers coated with PVP).^13^ However, on mixed samples, when flakes of different natures can stack together, this height strategy fails. Moreover, height classification can be controversial; for example, Liu et al. performed an AFM topography study of MoS_2_@PVP composites, and they proposed that the thickness of ≈2.7 nm belonged to a few-layered material instead of a thicker composite.^35^ But the authors did not clarify why the exfoliation method exclusively produces few-layer flakes of constant thickness and there is no trace of monolayers; hence, in our opinion, the formation of a homogeneous composite with a thickness of ≈2.7 nm would be more reliable.^13^

**Figure 4:**
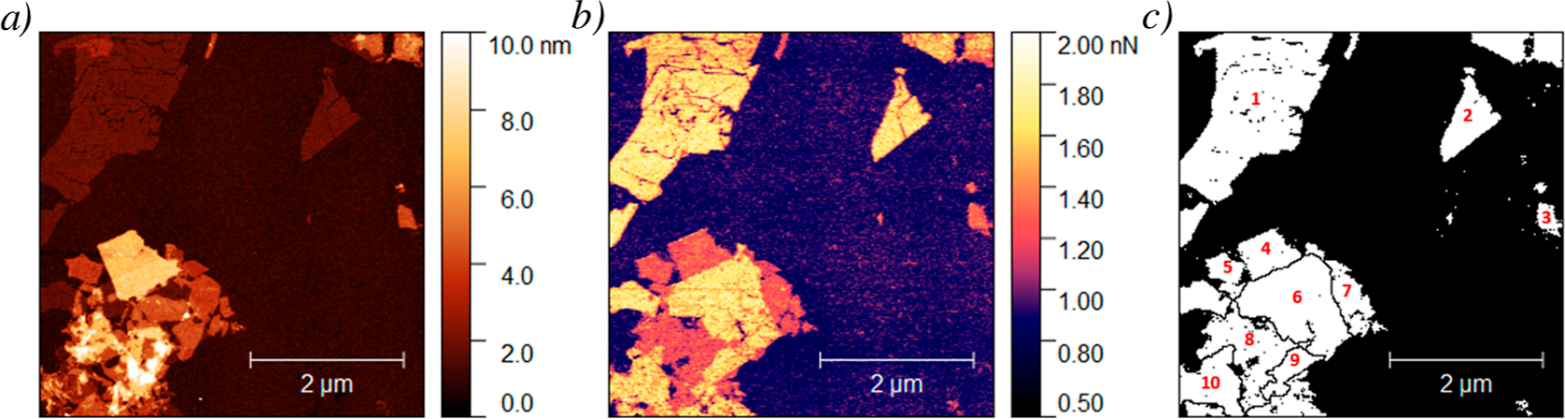
AFM images of a MnPS_3_@Mix sample obtained with a SAA probe: (a) topography channel, (b) adhesion channel, and (c) distribution of image areas for further study.

In the present study, we have used the RA parameters to analyze the top layer of several flakes individually, thus identifying in an unambiguous way the chemical nature of this top layer (H_2_O or PVP coating). Figure 4 depicts the results obtained for a MnPS_3_@Mix sample measured with a SAA probe. In Figure 4a, we observe the topography of a MnPS_3_@Mix sample, where flakes of different heights can be seen. In Figure 4b, we observe the adhesion signal for the above-mentioned sample. Interestingly, we can clearly distinguish between the two well-defined types of flakes according to this signal with well-distinguished boundaries. The distribution of the data in two populations is clearer when observing the RA versus height plotted in Figure 6. Hence, using these borders, different areas are selected (Figure 4c), and their parameters of height and adhesion are analyzed individually.

It is also worth mentioning that some flake boundaries are only detected when observing the adhesion channel and are almost invisible to the topography one (see the area labeled as number 6). This may indicate that a single MnPS_3_ monolayer adapts its shape to the material beneath, making it difficult to detect its size and shape only by topographic analysis. Meanwhile, the adhesion channel clearly depicts the flake’s boundaries. This effect highlights the power of this method to be sensitive to the outermost layer of the composite.

**Figure 5:**
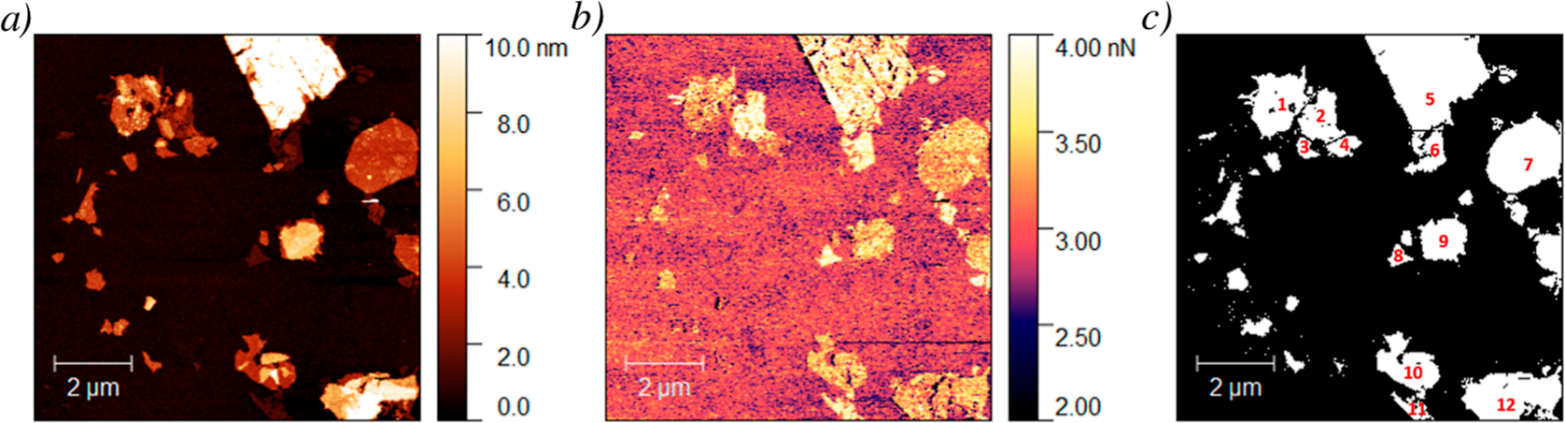
AFM images of a MnPS_3_@Mix sample obtained with a RTESPA-150 probe: (a) topography channel, (b) adhesion channel, and (c) distribution of image areas for further study.

**Figure 6:**
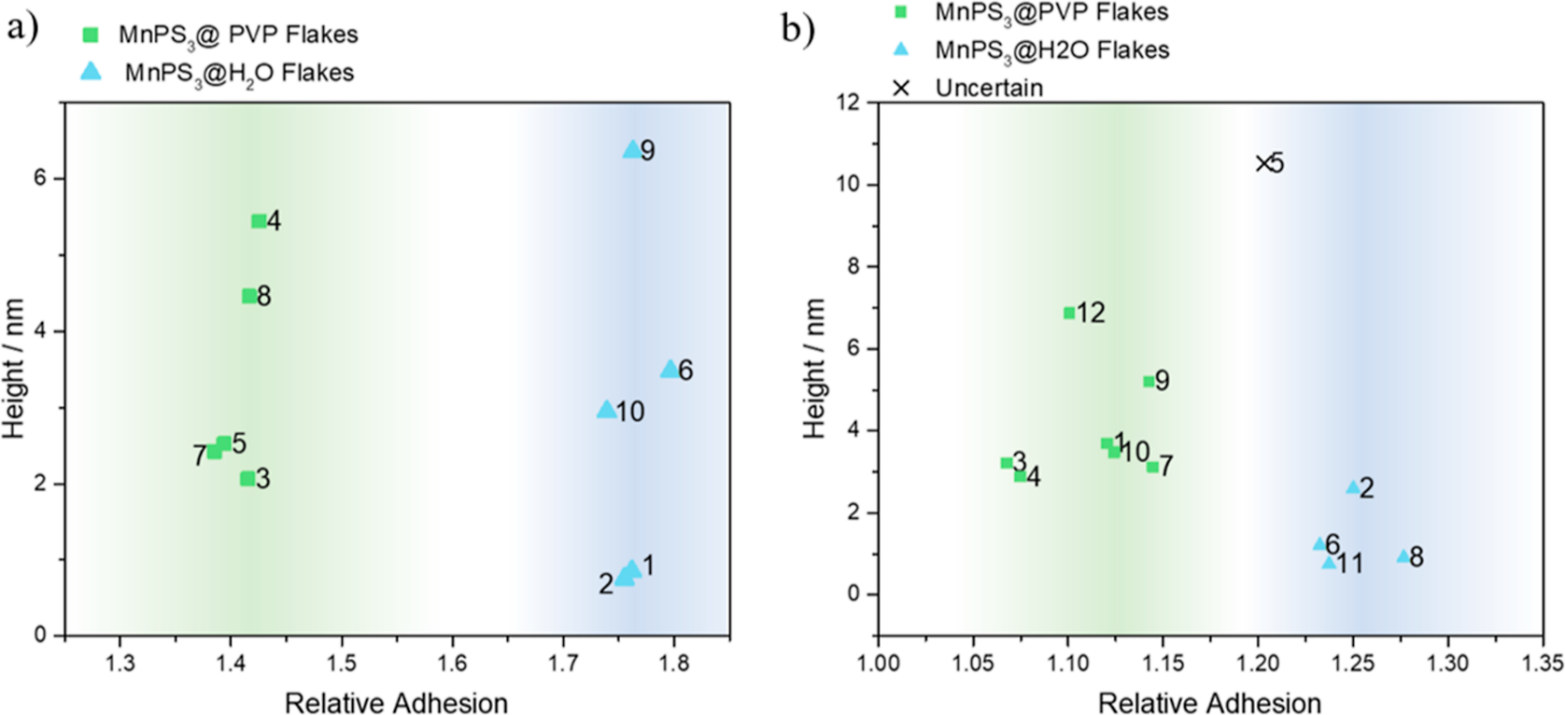
Cluster analysis of height vs RA of MnPS_3_@Mix samples. Images taken with the (a) SAA probe and (b) RTESPA-150 probe.

An identical analysis was conducted using a RTESPA-150 probe, and the results are presented in Figure 5. In this case, a similar result is obtained. Thus, the topography image is shown in Figure 5a, where layers and multilayers of different thicknesses are observed, whereas in Figure 5b, only two main adhesion contrasts are distinguished in the adhesion channel. In this case, it is difficult to differentiate between the two compounds at a glance. However, after analyzing the RA data, it is clear how these are arranged in the two populations (as will be discussed later and is depicted in Figure 6). Similar to the case before, these borders were exploited for drawing flake boundaries in Figure 5c and analyzing the height and RA values.

Finally, in Figure 6, the thickness versus RA values obtained for MnPS_3_@Mix samples with both probes are examined. When using SAA probes (Figure 6a), it is very easy to distinguish between two populations of data: a cluster of lower RA (1.2–1.4) and another one with higher RA (1.72–1.8). According to the prior testing of individual samples (Figure 2), we determined that the first group corresponds to PVP-covered areas, while the second group belongs to free MnPS_3_ flakes. With the RTESPA-150 probe (Figure 6b), the classification of data points may lead to a few uncertainties. It is possible to distinguish two populations of data between 1.0 and 1.14 and 1.20–1.27, which are slightly broader than the previous case, but differences between clusters are still evident. Just one flake may lead to uncertainty (flake 5). As was done for individual samples, the clusters in Figure 6 have been analyzed with the ANOVA test, resulting in values of RA being significantly different between both types of flakes (see Sections S6 and S7 for more details).

In order to test if MnPS_3_@PVP and MnPS_3_@H_2_O samples could be automatically classified based on their RA values, a cluster analysis was performed through the “K-means” method, which is an unsupervised classification algorithm extensively used in machine learning models. In this case, the algorithm suggests the exact classification result proposed in Figure 6. The script and fundamental description of the method are shown in Section S7. The results of this unsupervised analysis suggest that both probes are capable of distinguishing between both samples, with SAA being the one that provides the best results.

### 2.4 Study of Flakes of Different Natures

Chemical exfoliation methods often involve the presence of intercalating-charged agents to separate the layers. In this case, K^+^ and Li^+^ ions are used. Therefore, some electrostatic charges are created on the flakes, and their mechanical properties can be changed. Some techniques only allow the determination of an average value for the surface charge on the flakes. More information may be obtained using AFM and assessing adhesion parameters. In this case, these changes can be studied in detail with spatial resolution.

**Figure 7:**
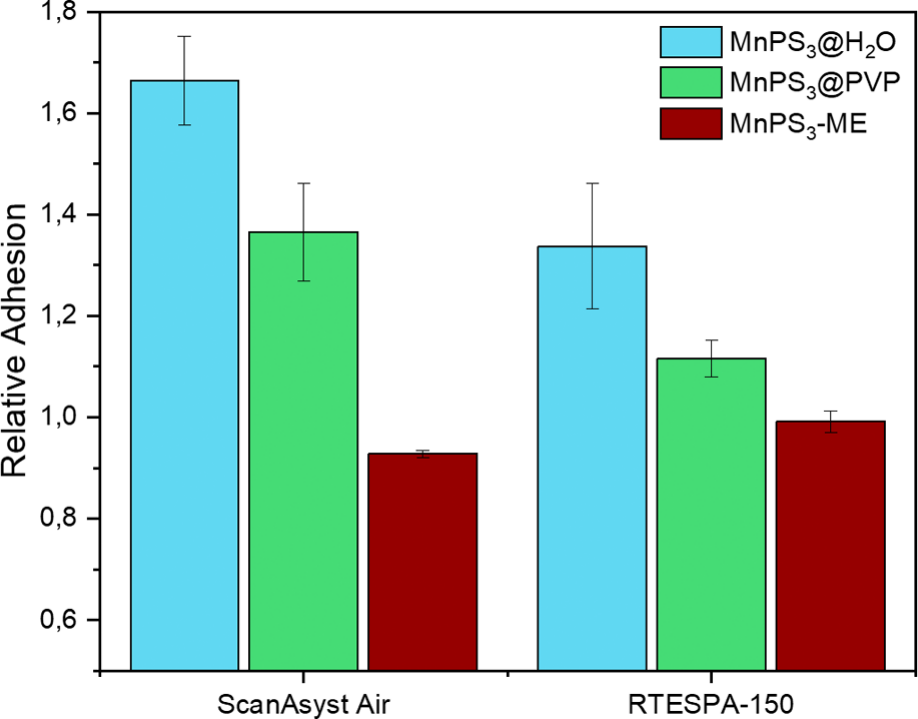
RA measured for MnPS_3_@H_2_O, MnPS_3_@PVP, and ME-MnPS_3_ with SAA and RTESPA-150 probes.

Here, we use MnPS_3_@H_2_O and MnPS_3_@PVP chemically exfoliated samples and compare their RA with those of a mechanically exfoliated sample (MnPS_3_-ME). For obtaining the highest RA contrast, SAA and RTESPA 150 probes were used. The results are plotted in Figure 7, and the AFM images analyzed for obtaining the data can be found in Section S8. These results show a clear decrease in the RA values for the three kinds of samples with both tips under study, i.e.:

RA(MnPS_3_@H_2_O) > RA (MnPS_3_@PVP) > RA(MnPS_3_-ME).

This observation indicates stronger tip–sample interactions when flakes are unprotected chemically exfoliated layers, in agreement with the larger number of defects and large surface charge in these layers.^13^

As was stated before, ambient humidity plays an important role in the probe-sample adhesion. This humidity creates a layer of water molecules on the surface of the samples, and when the AFM tip approaches the surface, a water meniscus is formed between the tip and the surface, which can modify probe-sample interaction.^36^ Hence, the adhesion measurement of the flakes can be an indirect indicator of the surface charges of the samples. Looking at the results, we can conclude that the less charged material is the mechanically exfoliated one. Between the two chemically exfoliated systems, MnPS_3_@PVP has a polymeric coating partially compensating MnPS_3_ charges, while in MnPS_3_@H_2_O, the layers are unprotected and present a higher superficial charge, as proven by these results. Therefore, it should be considered that adhesion results may vary depending on the humidity level of the environment. However, using RA can help minimize this effect to some extent. Nevertheless, the nature of the flake itself is the most important factor. Ultimately, to obtain the best results, it is recommended to avoid significant changes in environmental conditions.

Finally, it is interesting to note the higher relative errors yielded by RTESPA-150 than by SAA probes (Figure 2). This can be related to the fact that among probes with similar coatings, softer probes exert less force on the surface and are less degraded during measurements. Therefore, this could be the reason for the uncertainty in analyzing some areas in the mixed samples, as observed in the previous section (Figure 6).

Furthermore, intrigued by the role played by the 2Dm nature, we extended our study to MoS_2_ and performed an initial RA test on MoS_2_@H_2_O and MoS_2_@PVP. The obtained results, although preliminary, show a clear trend of stronger adhesion of MoS_2_@PVP layers, which is very close to that of the substrate (see Section S9). This fact is opposite the RA trend for MnPS_3_ samples, which supports the relevance of the chemical nature of the 2D material and simultaneously proves the usefulness of the RA method for distinguishing different materials.

## 3 Conclusions

The increasing interest in 2Dms is based not only on their unique properties but also on their potential applications in fields such as optoelectronics, medicine, and energy harvesting and storage. The chemical modification and molecular functionalization of 2Dms open these systems to a broader spectrum of possibilities; however, the proper characterization of these modifications is always very difficult. At a moment where simple and direct spatially resolved chemical and mechanical characterization of 2Dms has become a handicap for quick development of the field, we have shown that AFM adhesion measurements at ambient conditions can turn out to be a simple solution. The method presented can discern between two materials with similar topography but different chemical compositions on the surface. The probes used are quite common and commercially available, avoiding the need for any functionalization on the probe, which eases the characterization process and improves reproducibility. The use of low forces by the probe against the samples ensures less degradation of the sample and the probe and a higher level of surface sensitivity. It is important to point out that this method is sensitive only to the top layer of the material and not to the layers beneath it, giving the same result for a specific material despite the number of layers or the composition of the material under the top layer. Additionally, as it is an AFM-based method, it possesses a very high lateral resolution, on the order of a few nanometers, at least. This feature is ideal for determining the spatial distribution and the homogeneity of the chemical functionalization in the cutting-edge field of 2Dms. However, the weaknesses of this method could be identified as a lack of reproducibility due to variability in ambient conditions and modifications of the AFM probe apex during measurements. The first effect can be specifically minimized by controlling ambient humidity and temperature, while both are addressed by using RA instead of absolute adhesion, thereby overcoming the limitations related to reproducibility. Therefore, the normalization of adhesion data to produce RA values that are consistent between probes and sample thicknesses is a very remarkable result, and it potentially represents a step change in the interpretation and comparison of adhesion measurements. The issue that could be very difficult to overcome using the RA approach is the differentiation between surfaces with very close chemical compositions because the RA range of values for each material will overlap.

Finally, it has been shown that automated classification algorithms based on inferential statistics are very profitable tools to confirm and automate 2Dm characterization experiments. Prospects on applying artificial intelligence algorithms for image analysis and feature classification may be of great interest to the AFM scientific community.

## 4 Experimental Section

Sample preparation: The synthesis and exfoliation of MnPS_3_ flakes have been described in previous works.^13^ The result of this exfoliation procedure is suspensions of MnPS_3_ flakes in water or aqueous solutions of PVP (10 g/L). For removing unexfoliated material on further steps, these samples, called MnPS_3_@H_2_O and MnPS_3_@PVP respectively, were centrifuged at 3000 rpm for 15 min, and the supernatant was collected. In the case of MnPS_3_@PVP samples, the excess PVP was cleaned in water with three successive high-speed centrifuge cycles (10.000 rpm, 30′).

Once the suspensions were clean, they were suitable for depositing on the substrates. To do so, the suspensions were diluted to 1/10th their concentration in water. Subsequently, 10 μL of this suspension was spin-coated on a 1 × 1 cm^2^ Si/SiO_2_ substrate spinning at 50 rpm and allowed to spin for 60 s.

For preparing mixed samples, 10 μL of each suspension was added to the same substrate. In these cases, the MnPS_3_@PVP suspension was added in the first place to avoid the deposition of PVP molecules onto previously deposited MnPS_3_ flakes, which could lead to ambiguous results. This way, the MnPS_3_@PVP solution was spin-coated first and then MnPS_3_@H_2_O.

AFM analysis of the samples: All of the samples were analyzed using a Bruker Dimension Icon atomic force microscope in quantitative nanomechanical mode. Details of the probes used can be found in Table S1. Although manufacturers provide the nominal parameters for the probes (deflection sensitivity and force constant), the specific values for each one need to be assessed. Hence, the deflection sensitivity and force constant of the probe used were determined by performing force curves on a standard sapphire sample. Once the parameters are known, the samples can be measured. To do so, the system performs force curves on the area of analysis. The applied force (*i.e*., PFS) is set as low as possible to ensure good indentation (≈1 nm) and a stable measurement. For most of the probes, the necessary force was 0.5–1.0 nN, but in the case of Tap 300-G, a higher PFS was needed (≈25 nN) to obtain a stable force curve and an acceptable measurement.

Later, SAA and RTESPA-150 probes were used to study MnPS_3_@Mix samples and detect the different flakes on the surface.

Mechanically exfoliated MnPS_3_: Highly crystalline MnPS_3_ was exfoliated through the well-known Scotch-tape method and deposited onto Si/SiO_2_ substrates. Here, a piece of adhesive tape was stacked to the 2Dm crystals and peeled off. The procedure was repeated several times until the 2Dm was thin enough. Finally, the Scotch tape with 2Dm flakes was stamped on a Si/SiO_2_ substrate. The obtained flakes were analyzed with SAA and RTESPA-150 probes using the same procedure as that for the rest of the samples.

For transforming raw data into topography and adhesion images and obtaining the height and adhesion values, Gwyddion 2.53 open-source software was employed.

## Supporting Information Available

Brief explanation of cantilever movement during force–distance curve measurements; AFM probe selection experiments; reproducibility study of AFM probes; adhesion–thickness analysis; analysis of the influence of the applied PFS on the adhesion response of the probe; statistical analysis of RA for MnPS_3_@H_2_O and MnPS_3_@PVP samples; classification process; comparison with mechanically exfoliated flakes; and analysis of MoS_2_ samples (PDF)

## Abbreviations

2Dms: 2D materials
AFM: atomic force microscopy
PVP: polyvinylpyrrolidone
RA: relative adhesion
PFS: PeakForce set point
SAA: ScanAsyst Air
