# Supplementary material for: Atomic Force Microscopy beyond Topography: Chemical Sensing of 2D Material Surfaces through Adhesion Measurements
Source: ACS Appl Mater Interfaces. 2024 Apr 3;16(15):19711–9. doi: 10.1021/acsami.3c19254 (PMC11040525; doi:10.1021/acsami.3c19254)
Supplement: Supplementary file 1 — am3c19254_si_001.pdf [file am3c19254_si_001.pdf]

Supporting Information

# Atomic Force Microscopy Beyond Topography: Chemical sensing of 2D materials surface through adhesion measurements.

*Isaac Brotons-Alcázar, Jason. S. Terreblanche, Silvia Giménez-Santamarina, Gerliz M. Gutiérrez-Finol, Karl S. Ryder*, Alicia Forment-Aliaga*, and Eugenio Coronado*

I. Brotons-Alcázar, Silvia Giménez-Santamarina, Gerliz M. Gutiérrez-Finol, A. Forment-Aliaga, E. Coronado.

Instituto de Ciencia Molecular (ICMol), Universitat de València, C/ Catedrático José Beltrán Martínez, 2, 46980 Paterna, Spain.

Jason. S. Terreblanche, Karl S. Ryder

Center for Sustainable Materials Processing, School of Chemistry, University of Leicester, University Road, Leicester, LE1 7RH, UK

**Corresponding Authors**

*Alicia Forment-Aliaga. alicia.forment@uv.es

* Karl S. Ryder. ksr7@leicester.ac.uk

# Table of Contents

**1. Cantilever movement during force-distance curves measurements, a brief explanation.** 3

**2. AFM probe selection experiments.** 4

**3. Reproducibility study of AFM probes.** 12

**4. Adhesion-Thickness analysis.** 18

**5. Influence of the applied PeakForce Setpoint on the adhesion response of the probe.** 21

**6. Statistical analysis of RA for $MnPS_3$@$H_2O$ and $MnPS_3$@PVP samples** 25

**7. Classification process.** 30

**8. Comparison with mechanically exfoliated flakes.** 32

**9. Analysis of $MoS_2$ samples** 34

**10. References** 37

## 1. Cantilever movement during force-distance curves measurements, a brief explanation.

Regarding the Figure 1 in the main text, we can describe the movement of an AFM probe while measuring: When a free-standing AFM cantilever (I) gets its probe close enough to the surface, an attractive force with the substrate appears due to long range interactions (e.g.: van der Waals ones). This makes the cantilever to bend towards the substrate snaping into it (II). If the approaching step continues, the cantilever experiences repulsive forces and bends in the opposite direction while the tip indents the sample (III). In the detaching step (IV), the probe experiences significant attraction due to the superficial adhesion forces of the sample to the tip while bending more than in the previous approach step II. The value of the adhesion force is the depth of the well observed in the force-distance curve during this step (red trace in figure 1b in the main text). Finally, the probe is released from the tension so it can approach again (V).

## 2. AFM probe selection experiments.

In Table S1 it is possible to find all the main parameters for the probes used in this work. Note that these are the nominal values, the calibration made prior to the measurements allows to assess the specific resonance frequency and force constant for the specific probe used in each case. More information about the probes can be found free of charge on the next websites:

https://www.brukerafmprobes.com/ (For SCM-PIC, RTESPA-150, ScanAsyst Air and NPG-10A probes) Note that SCM-PIC has been replaced by SCM-PIC V2.

https://www.budgetsensors.com/tapping-mode-afm-probe-tap300 (For Tap 300-G).

**Table S1.-** List of probes used for this study and nominal parameters of each one.

| Probe | Force constant [$N \cdot m^{-1}$] | Tip radius [nm] | Resonance frequency [kHz] | Coating |
|---|---|---|---|---|
| SCM-PIC | 0.2 | 25 | 13 | Pt/Ir |
| Tap300-G | 40 | 10 | 300 | - |
| RTESPA-150 | 5 | 8 | 150 | - |
| ScanAsyst Air | 0.4 | 2 | 70 | - |
| NPG-10A[a)] | 0.35 | 30 | 65 | Au |

[a)]NPG-10 is a cantilever with four probes. “A” probe has been used in all cases.

AFM images of $MnPS_3$@$H_2O$ and $MnPS_3$@PVP used for probe study and selection are displayed below. Two images of each sample were recorded with each probe for obtaining a mean value in each case. For the Adhesion-Thickness study, (ESI Section 4) the data on each 2D flake is considered individually. For simplicity, this close analysis has been performed only in the first image of each set.

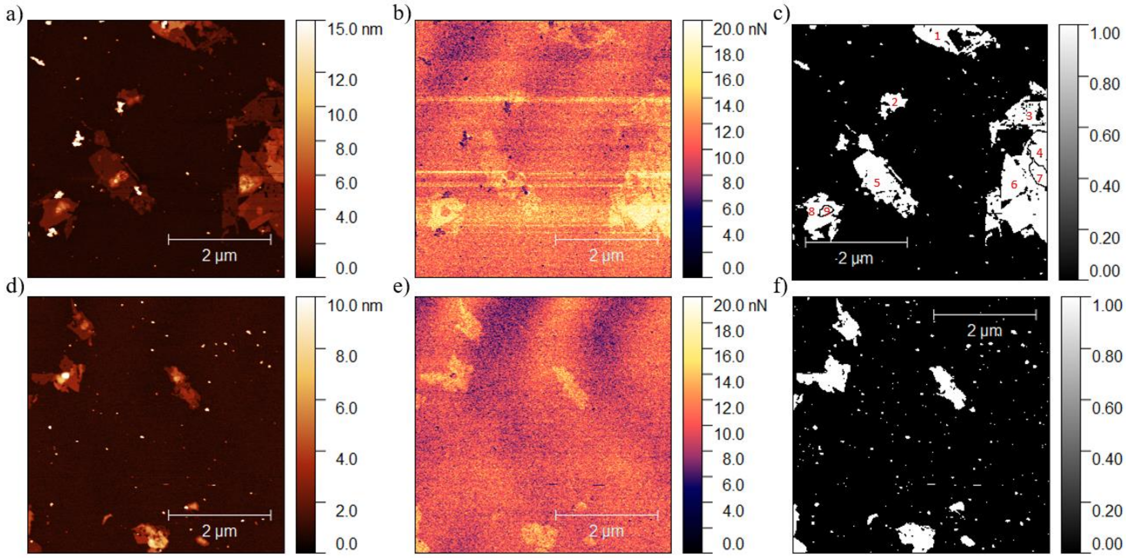


**Figure S1.** AFM images taken with Tap 300G probe on $MnPS_3$@$H_2O$ samples. First sample: a) topography channel, b) adhesion signal and c) areas selected for further analysis. Second Sample: d) topography channel, e) adhesion signal and f) area of the image considered for the mean adhesion on 2Dms.

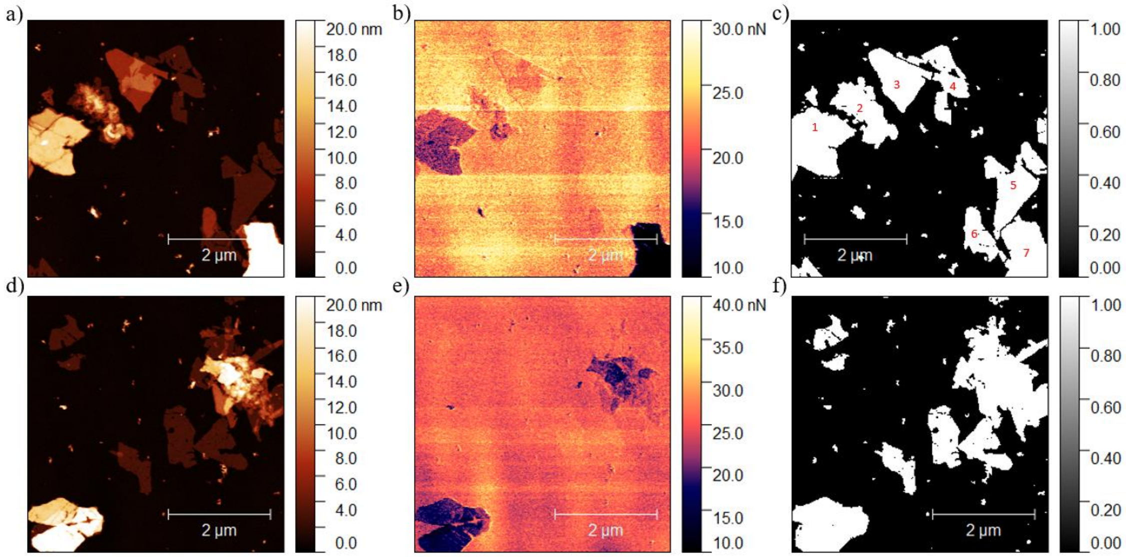


**Figure S2.** AFM images taken with Tap 300G probe on $MnPS_3$@PVP samples. First sample: a) topography channel, b) adhesion signal and c) areas selected for further analysis. Second Sample: d) topography channel, e) adhesion signal and f) area of the image considered for the mean adhesion on 2Dms.

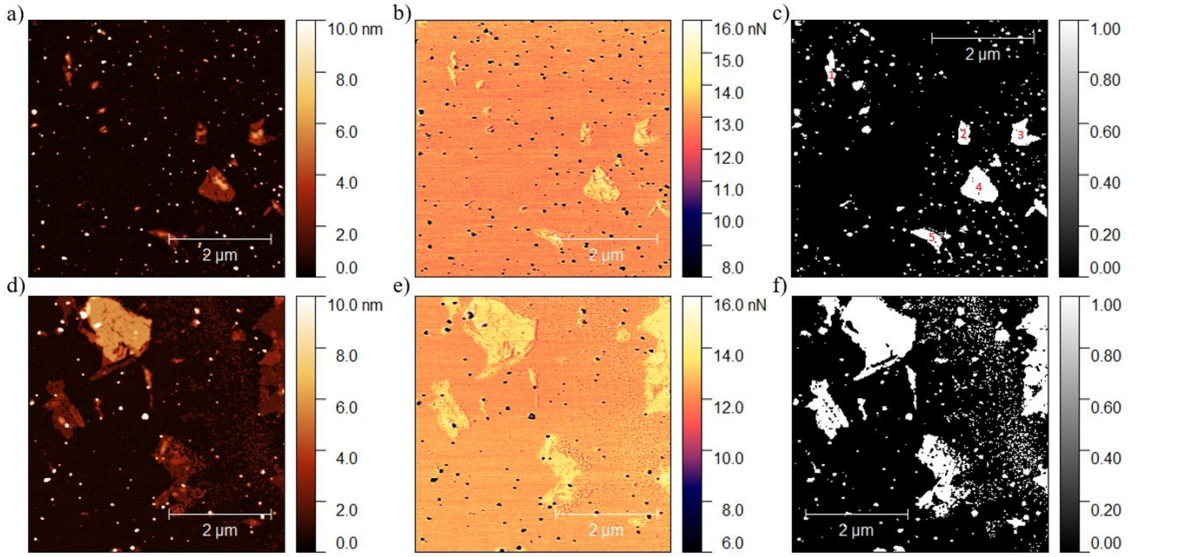


**Figure S3.** AFM images taken with NPG-10A probe on $MnPS_3@H_2O$ samples. First sample: a) topography channel, b) adhesion signal and c) areas selected for further analysis. Second Sample: d) topography channel, e) adhesion signal and f) area of the image considered for the mean adhesion on 2Dms.

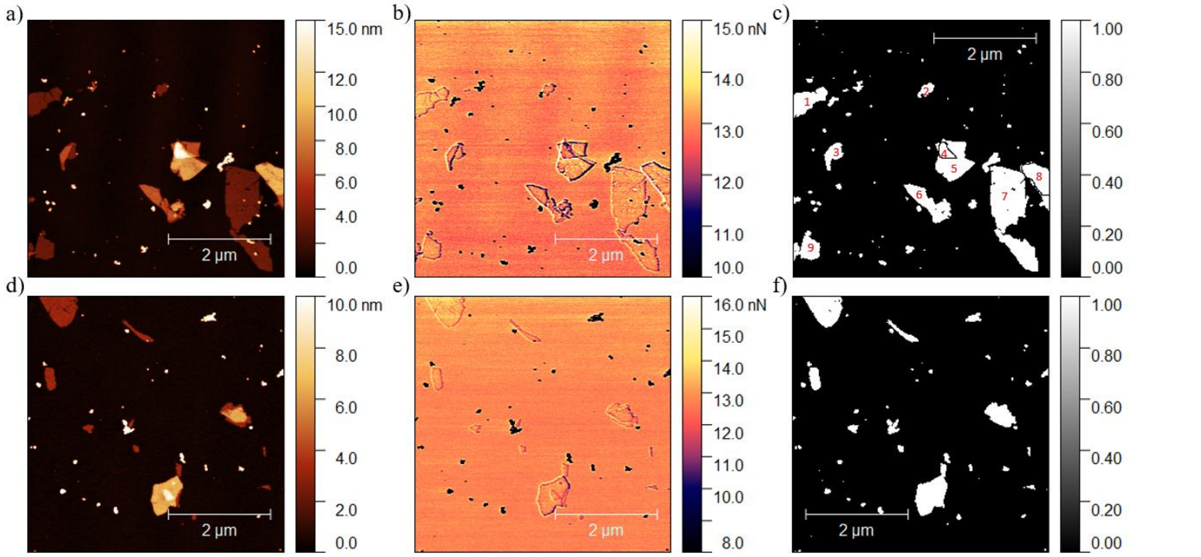


**Figure S4.** AFM images taken with NPG-10A probe on $MnPS_3$@PVP samples. First sample: a) topography channel, b) adhesion signal and c) areas selected for further analysis. Second Sample: d) topography channel, e) adhesion signal and f) area of the image considered for the mean adhesion on 2Dms.

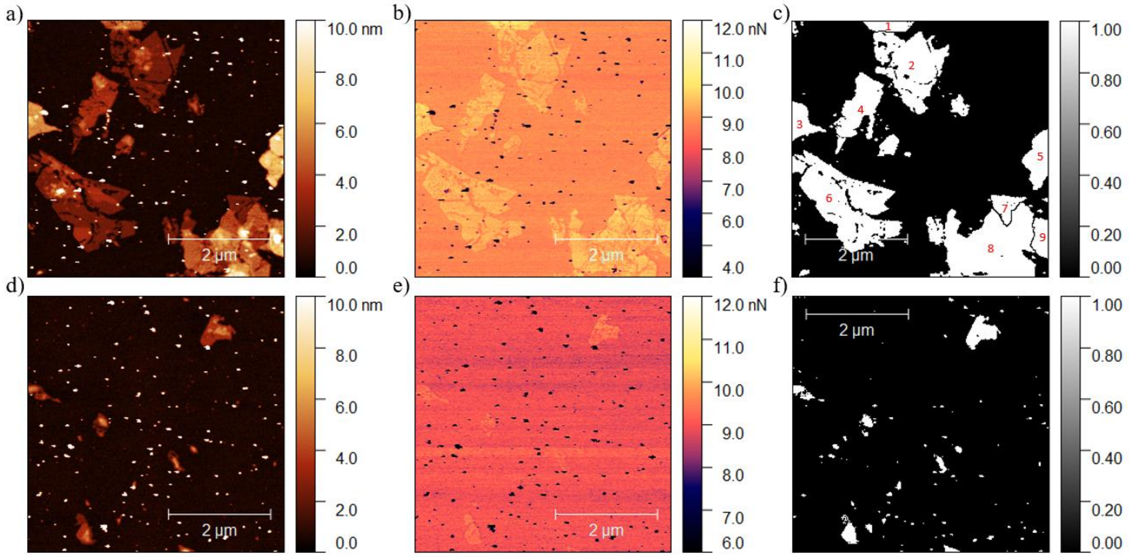


**Figure S5.** AFM images taken with SCM-PIC probe on $MnPS_3@H_2O$ samples. First sample: a) topography channel, b) adhesion signal and c) areas selected for further analysis. Second Sample: d) topography channel, e) adhesion signal and f) area of the image considered for the mean adhesion on 2Dms.

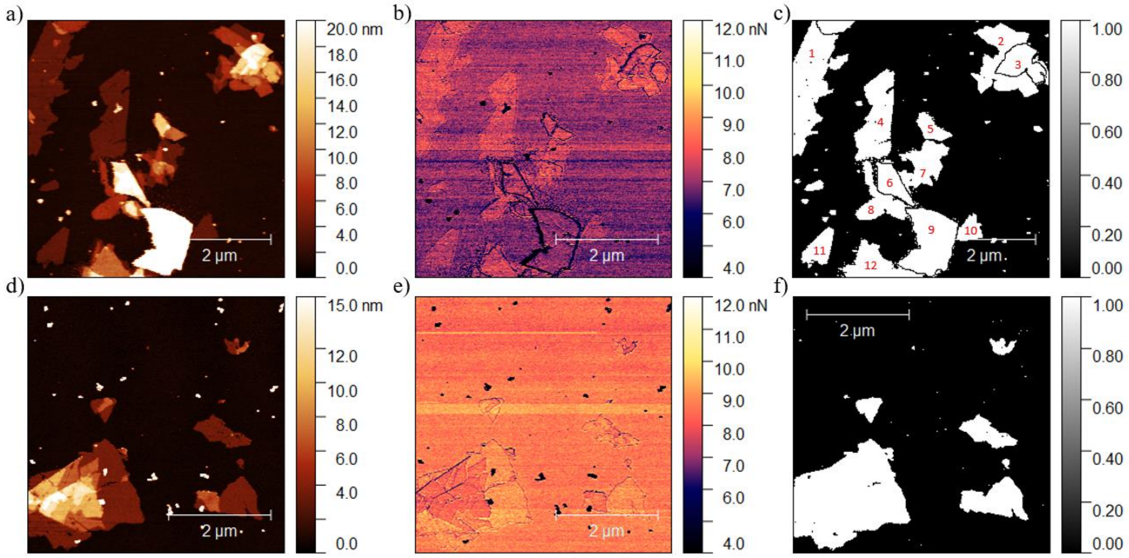


**Figure S6.** AFM images taken with SCM-PIC probe on $MnPS_3$@PVP samples. First sample: a) topography channel, b) adhesion signal and c) areas selected for further analysis. Second Sample: d) topography channel, e) adhesion signal and f) area of the image considered for the mean adhesion on 2Dms.

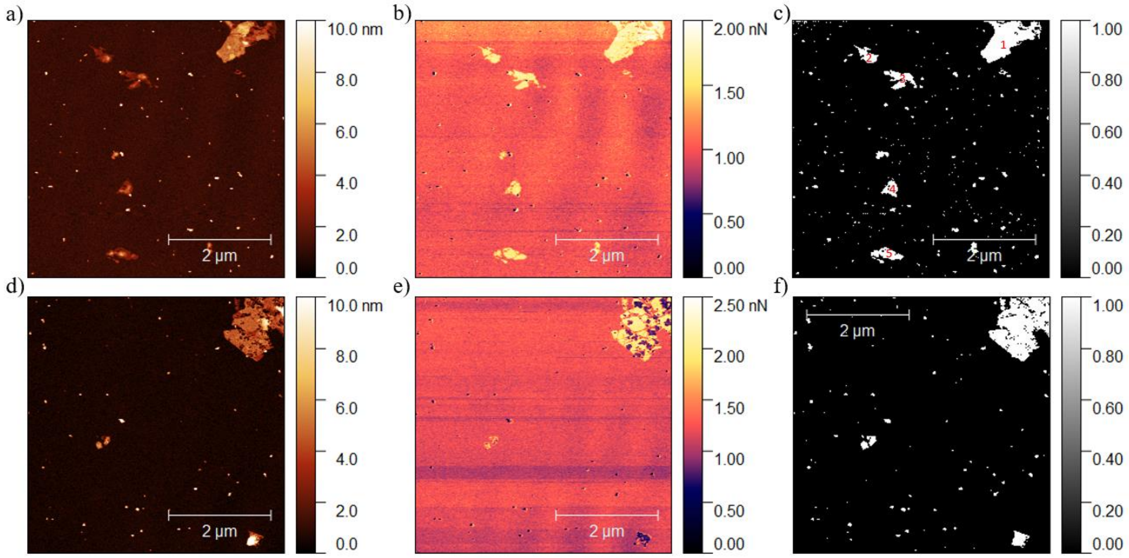


**Figure S7.** AFM images taken with RTESPA-150 probe on $MnPS_3$@$H_2O$ samples. First sample: a) topography channel, b) adhesion signal and c) areas selected for further analysis. Second Sample: d) topography channel, e) adhesion signal and f) area of the image considered for the mean adhesion on 2Dms.

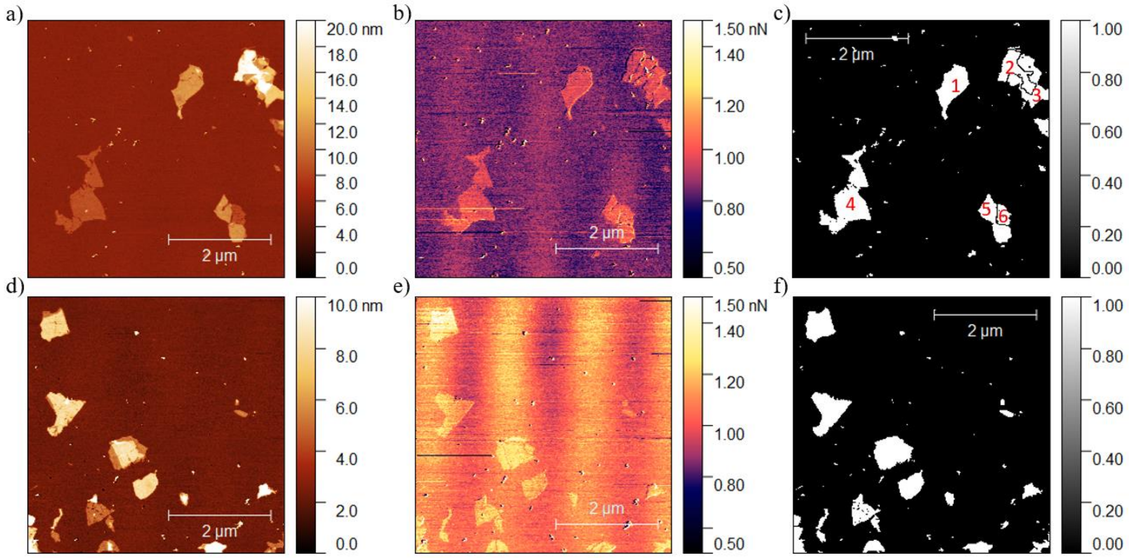


**Figure S8.** AFM images taken with RTESPA-150 probe on $MnPS_3$@PVP samples. First sample: a) topography channel, b) adhesion signal and c) areas selected for further analysis. Second Sample: d) topography channel, e) adhesion signal and f) area of the image considered for the mean adhesion on 2Dms.

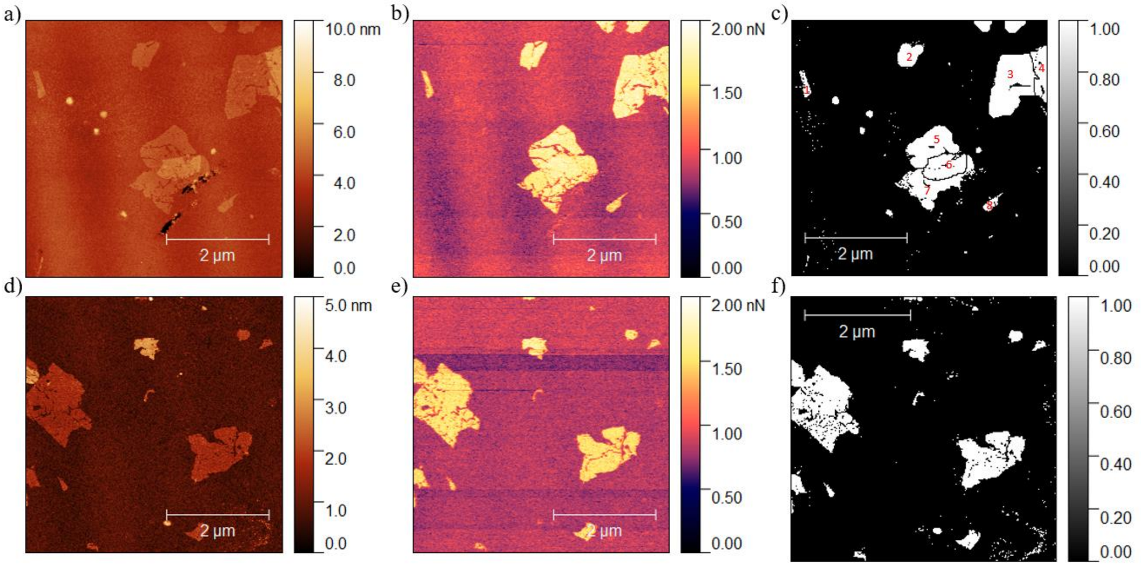


**Figure S9.** AFM images taken with ScanAsyst Air probe on $MnPS_3$@$H_2O$ samples. First sample: a) topography channel, b) adhesion signal and c) areas selected for further analysis. Second Sample: d) topography channel, e) adhesion signal and f) area of the image considered for the mean adhesion on 2Dms.

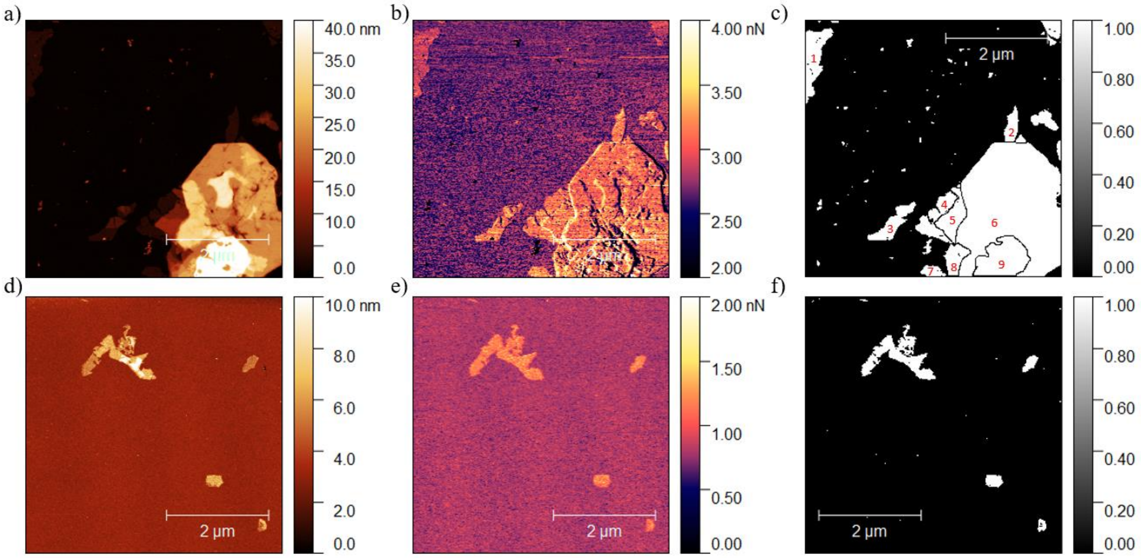


**Figure S10.** AFM images taken with ScanAsyst Air probe on $MnPS_3$@PVP samples. First sample: a) topography channel, b) adhesion signal and c) areas selected for further analysis. Second Sample: d) topography channel, e) adhesion signal and f) area of the image considered for the mean adhesion on 2Dms.

Regarding the metallic-coated probes (SCM-PIC and NPG-10A) the status of the probe was analysed after their utilisation. SEM imaging was done with EDX mapping, the results are depicted in Figures S11-S12.

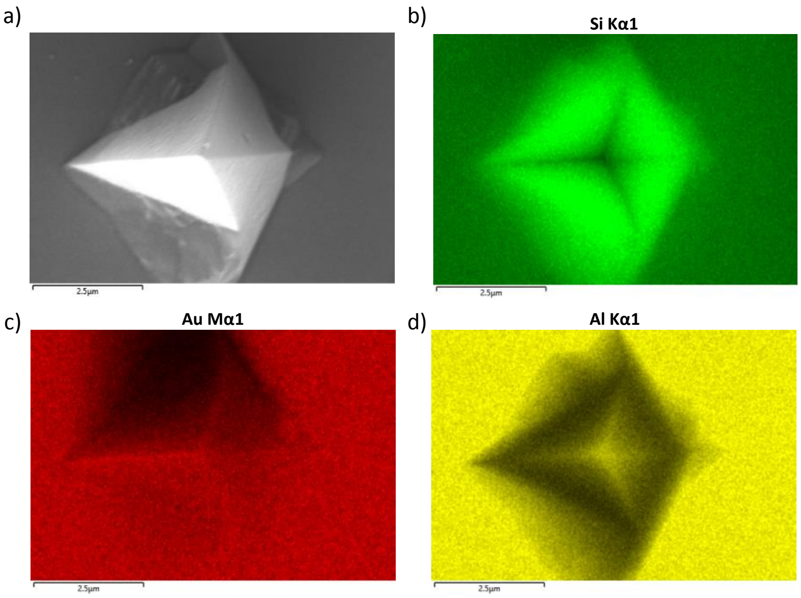


**Figure S11** SEM imaging and elemental analysis of a NPG-10A probe after using for AFM measurements. a) microelectronic image, b) Si element mapping, c) Au element mapping, and d) Al element mapping.

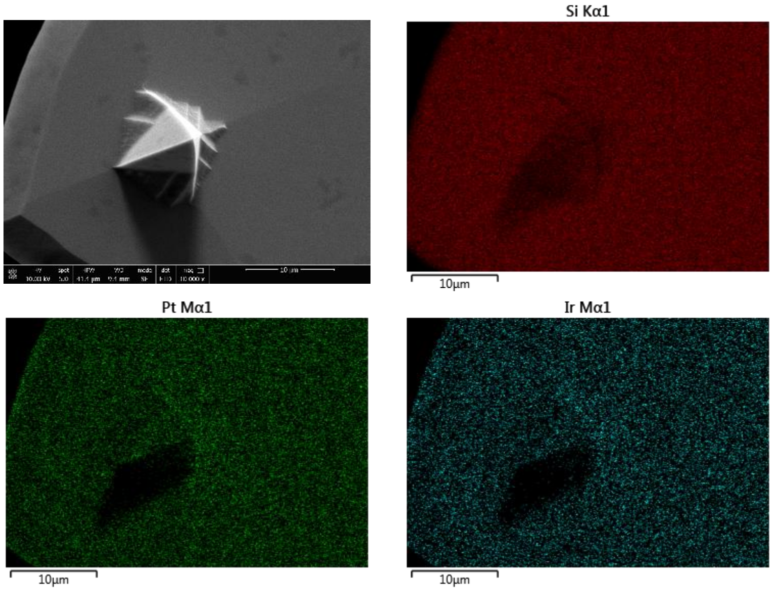


**Figure S12** SEM imaging and elemental analysis of a SCM-PIC probe after using for AFM measurements. a) microelectronic image, b) Si element mapping, c) Pt element mapping, and d) Ir element mapping.

As can be seen, it is not possible to appreciate any degradation or peeling-off of the coating. Moreover, the mapping signal of Au for NPG 10A, and Pt/Ir for SCM-PIC is homogenous all over the probe.

### 3. Reproducibility study of AFM probes.

A comparison between five different probes is discussed in the main text. A similar analysis was conducted with four ScanAsyst Air probes to evaluate the reproducibility of the method using different probes in different days and the results are depicted in Figure S13. In Table S2 is possible to compare the dispersibility of the data using either Adhesion or RA.

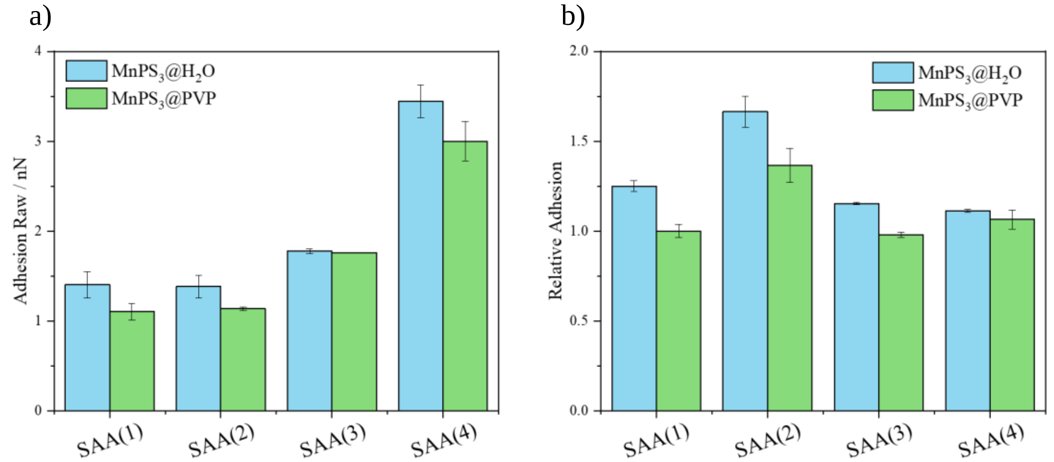


**Figure S13.** a) Raw Adhesion data of $MnPS_3$@$H_2O$ and $MnPS_3$@PVP samples obtained using four ScanAsyst Air (SAA) probes. b) RA obtained for the same samples.

The variation percentage of the data shown is analysed in Table S2, comparing the result obtained for Raw adhesion and RA. The analysis reveals how the use of RA instead of raw adhesion reduces the variation percentage of the data from ca. 50% to 15-20%. This is the expected effect, as RA considers in some way the status of the probe in each measurement.

**Table S2.** Variation percentage (%RSD) for the mean Raw Adhesion and RA obtained between all the ScanAsyst Air probes used.

| Data | %RSD for $MnPS_3$@$H_2O$ | %RSD for $MnPS_3$@PVP |
|---|---|---|
| Raw Adhesion mean | 48.8% | 50.6% |
| Relative Adhesion mean | 19.5% | 16.25% |

All the data analysed in Figure S13 and Table S2 was extracted from the following images (Figure S14-S21).

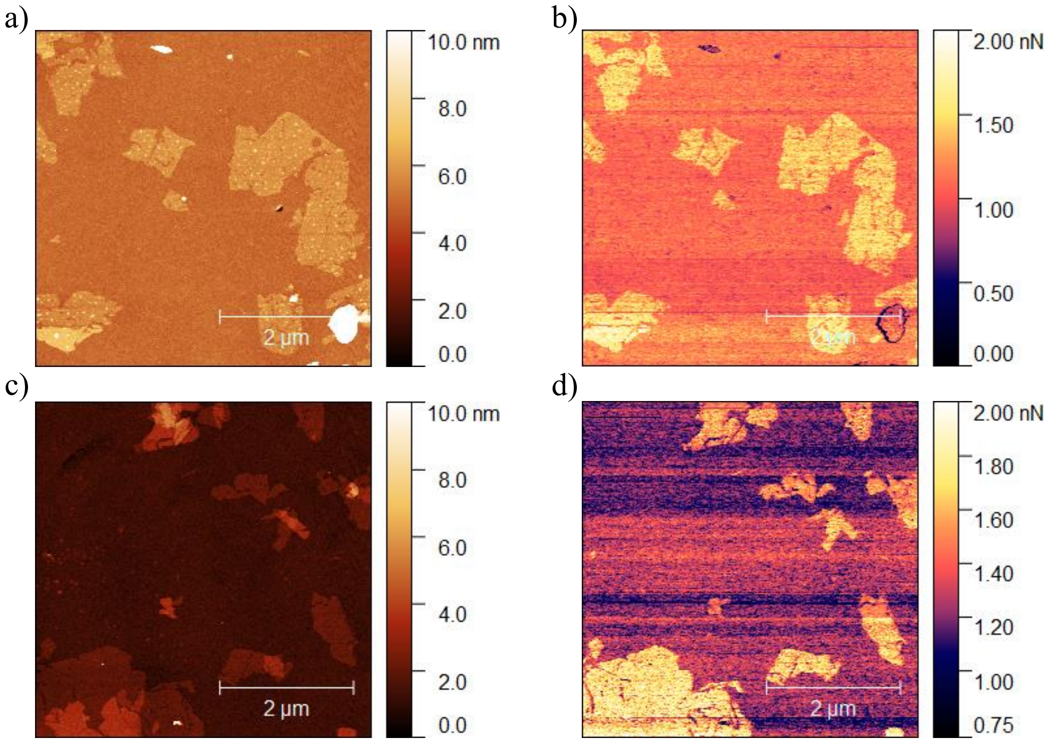


**Figure S14** Topography a), c) and Adhesion b), d) images measured with SAA(1) on $MnPS_3$@$H_2O$ samples for collecting the data plotted in Figure S13.

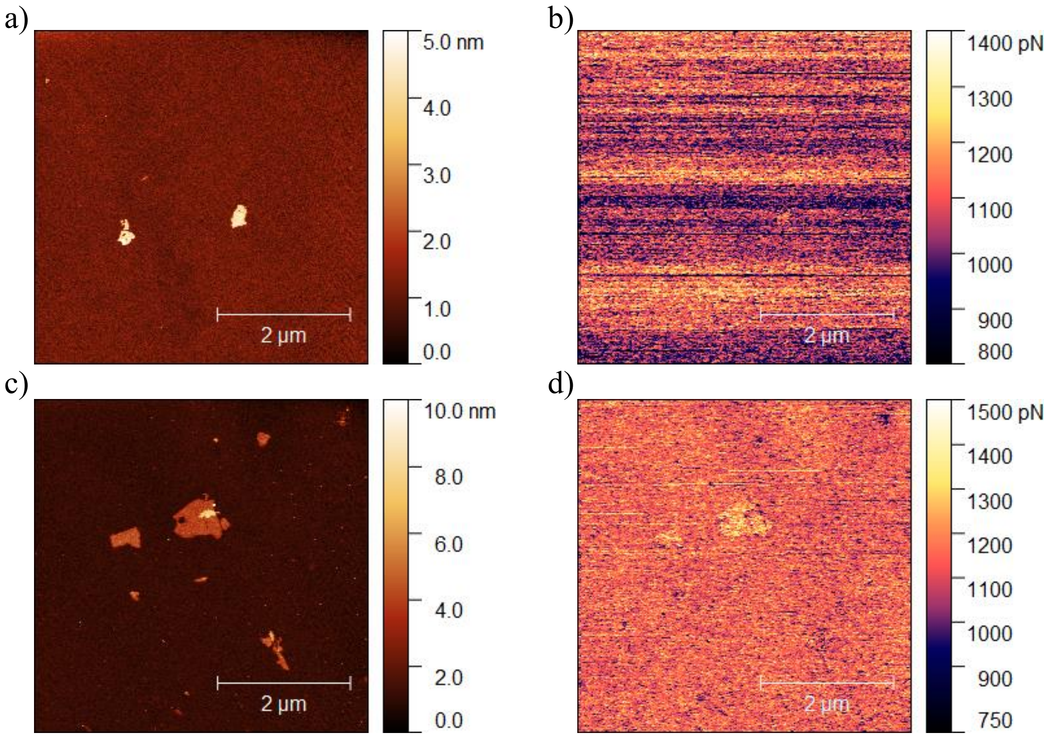


**Figure S15** Topography a), c) and Adhesion b), d) images measured with SAA(1) on $MnPS_3$@PVP samples for collecting the data plotted in Figure S13

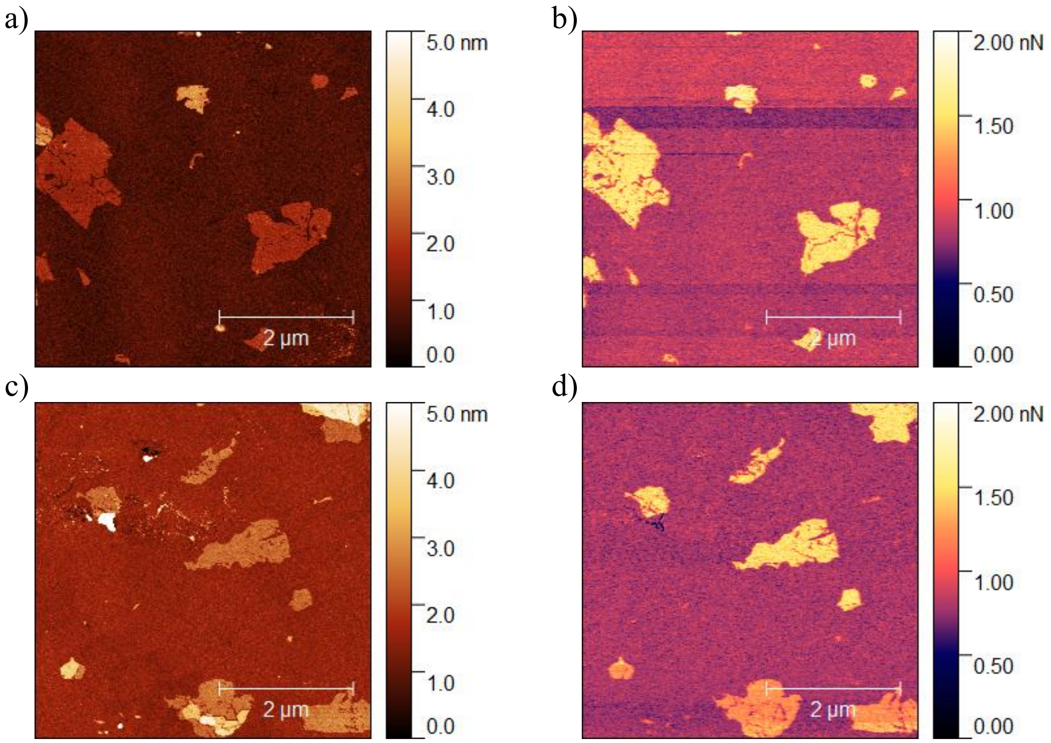


**Figure S16** Topography a), c) and Adhesion b), d) images measured with SAA(2) on $MnPS_3$@$H_2O$ samples for collecting the data plotted in Figure S13

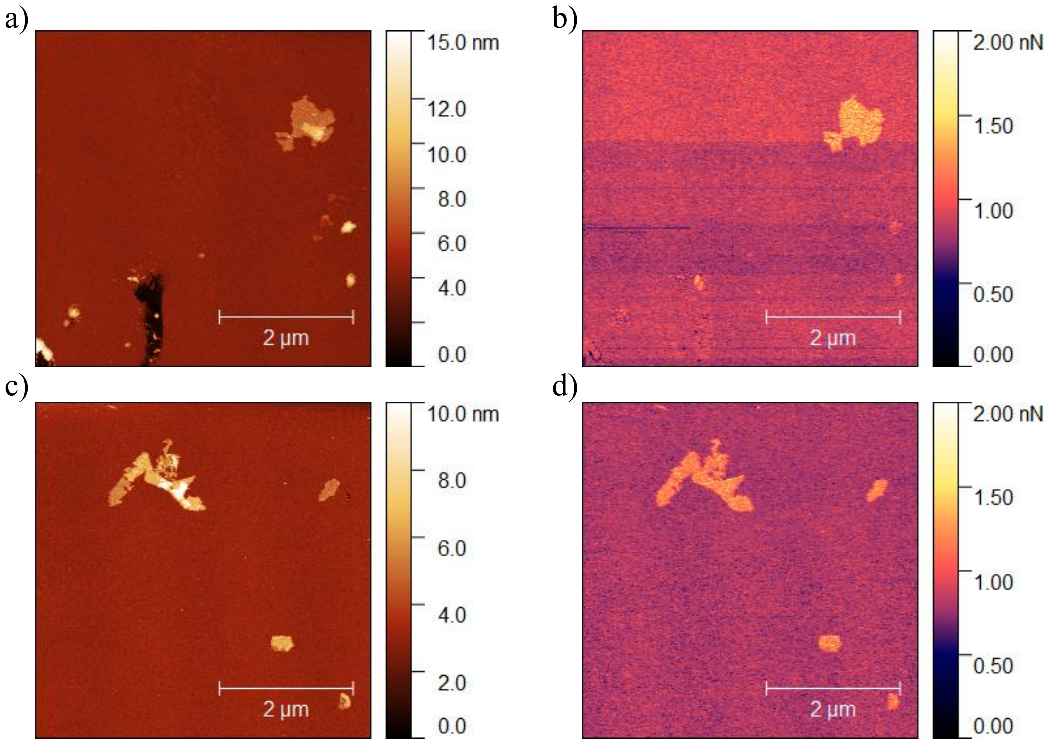


**Figure S17** Topography a), c) and Adhesion b), d) images measured with SAA(2) on $MnPS_3$@PVP samples for collecting the data plotted in Figure S13

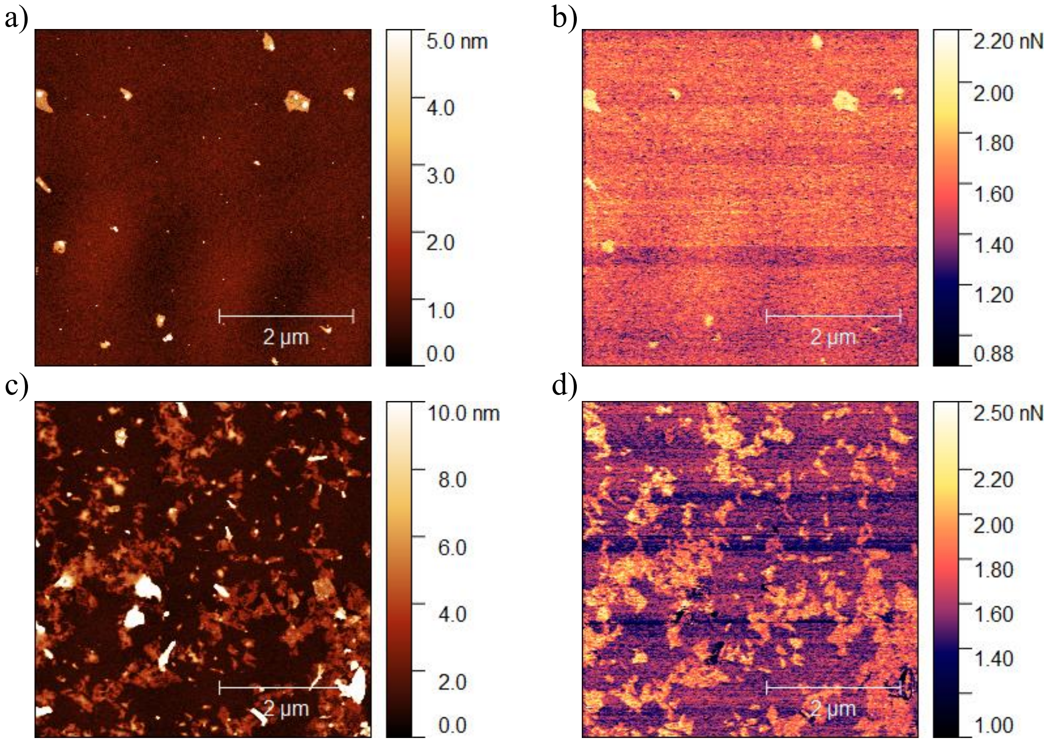


**Figure S18** Topography a), c) and Adhesion b), d) images measured with SAA(3) on $MnPS_3$@$H_2O$ samples for collecting the data plotted in Figure S13

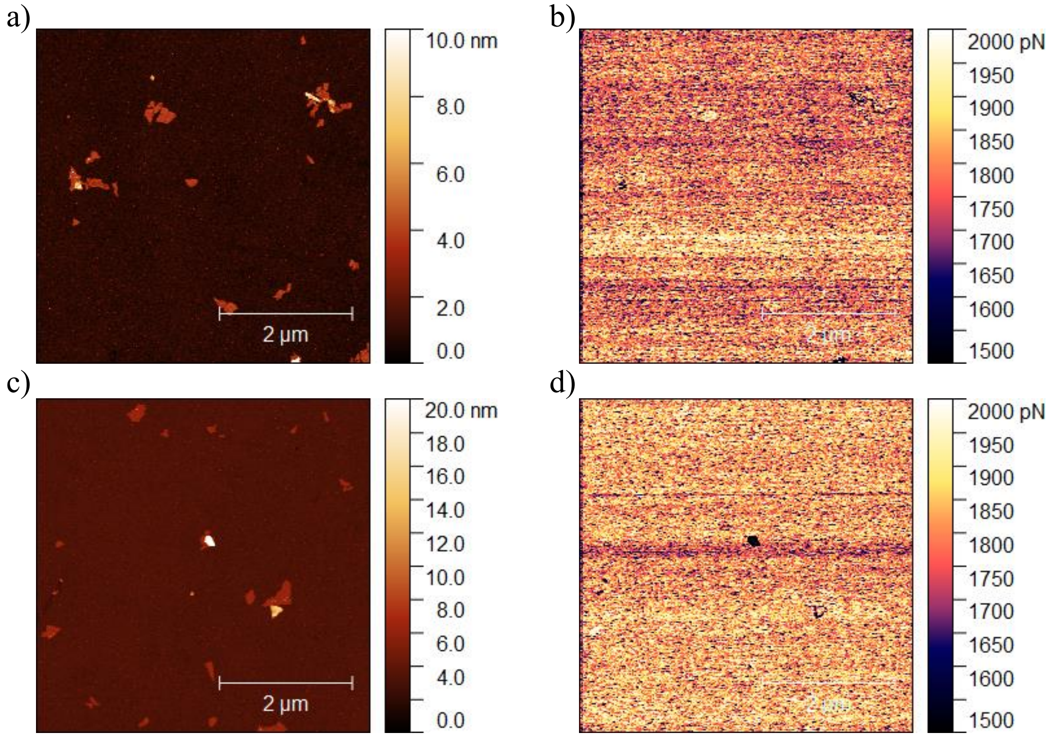


**Figure S19** Topography a), c) and Adhesion b), d) images measured with SAA(3) on $MnPS_3$@PVP samples for collecting the data plotted in Figure S13

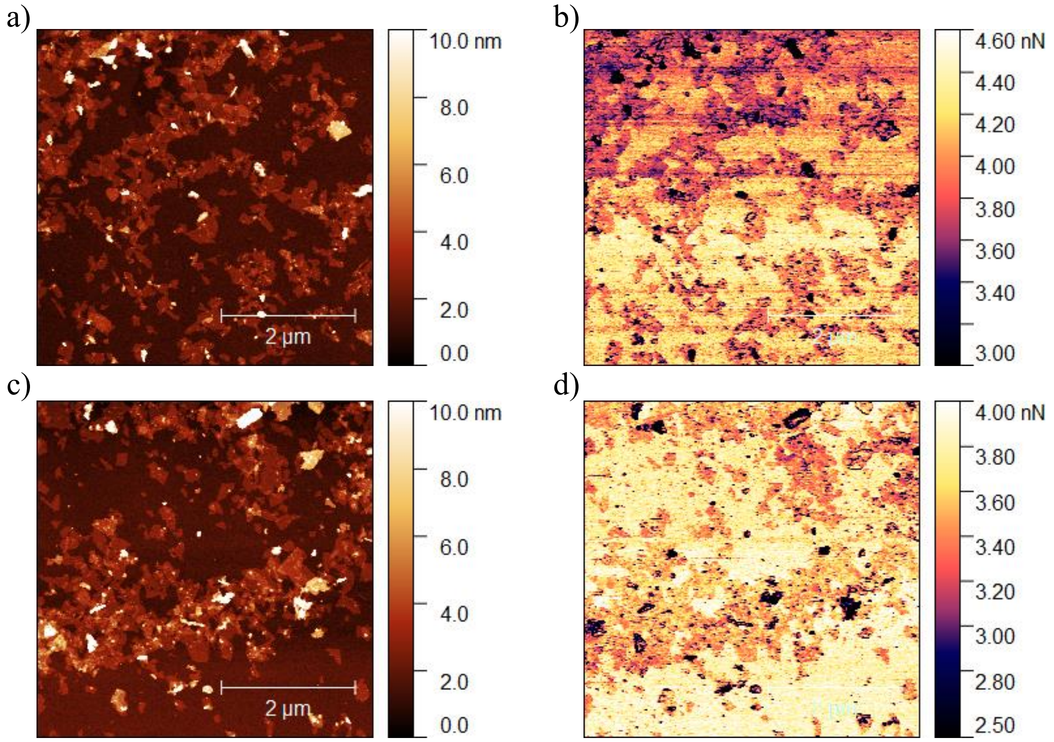


**Figure S20** Topography a), c) and Adhesion b), d) images measured with SAA(4) on $MnPS_3$@$H_2O$ samples for collecting the data plotted in Figure S13

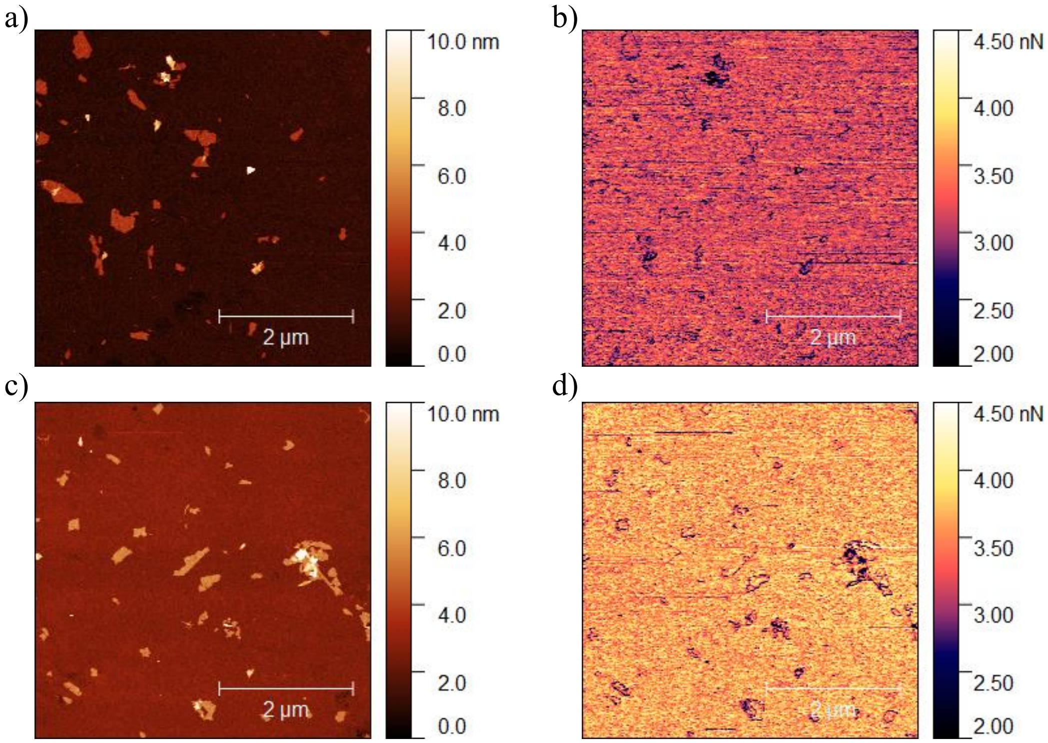


**Figure S21** Topography a), c) and Adhesion b), d) images measured with SAA(4) on $MnPS_3$@PVP samples for collecting the data plotted in Figure S13

**4. Adhesion-Thickness analysis.**

As has been described in the main text. The possible effect of the sample height on the adhesion channel was studied. To do so, several areas of AFM pictures taken with each probe have been selected and analyzed as can be observed in Figures S1-S10c) pictures of each one. Mean thickness and adhesion data for each area on these pictures is shown in the next tables (Table S3-S8)

**Table S3.** Mean height and adhesion for each area shown in Figure S1c and S2c for the data obtained with Tap 300G probe.

| | $MnPS_3$@$H_2O$ | | $MnPS_3$@PVP | |
|---|---|---|---|---|
| Area | Thickness [nm] | Adhesion [nN] | Thickness [nm] | Adhesion [nN] |
| 1 | 3.09 | 11.10 | 12.42 | 18.70 |
| 2 | 3.41 | 11.41 | 6.19 | 22.43 |
| 3 | 1.30 | 12.36 | 6.17 | 22.42 |
| 4 | 3.59 | 12.39 | 2.41 | 23.31 |
| 5 | 2.01 | 12.27 | 2.28 | 23.97 |
| 6 | 2.34 | 14.63 | 3.10 | 22.14 |
| 7 | 5.03 | 14.49 | 32.86 | 11.36 |
| 8 | 3.14 | 13.39 | - | - |
| 9 | 5.90 | 13.79 | - | - |

**Table S4.** Mean height and adhesion for each area shown in Figure S3c and S4c for the data obtained with NPG-10A probe.

| | $MnPS_3@H_2O$ | | $MnPS_3@PVP$ | |
|---|---|---|---|---|
| Area | Thickness [nm] | Adhesion [nN] | Thickness [nm] | Adhesion [nN] |
| 1 | 2.12 | 12.99 | 2.49 | 13.09 |
| 2 | 1.87 | 12.97 | 3.23 | 11.72 |
| 3 | 2.37 | 13.45 | 3.86 | 12.38 |
| 4 | 2.47 | 13.43 | 13.14 | 12.47 |
| 5 | 1.98 | 13.18 | 6.54 | 12.78 |
| 6 | - | - | 4.94 | 12.46 |
| 7 | - | - | 2.68 | 12.56 |
| 8 | - | - | 8.75 | 12.99 |
| 9 | - | - | 2.50 | 12.70 |

**Table S5.** Mean height and adhesion for each area shown in Figure S5c and S6c for the data obtained with SCM-PIC probe.

| | $MnPS_3@H_2O$ | | $MnPS_3@PVP$ | |
|---|---|---|---|---|
| Area | Thickness [nm] | Adhesion [nN] | Thickness [nm] | Adhesion [nN] |
| 1 | 3.51 | 9.70 | 2.59 | 7.62 |
| 2 | 1.76 | 9.12 | 5.02 | 7.62 |
| 3 | 5.03 | 9.29 | 14.36 | 7.30 |
| 4 | 2.45 | 9.12 | 2.66 | 7.49 |
| 5 | 5.92 | 8.97 | 9.06 | 6.95 |
| 6 | 2.15 | 9.12 | 16.84 | 6.75 |
| 7 | 1.54 | 9.27 | 4.58 | 7.23 |
| 8 | 3.48 | 9.23 | 6.28 | 6.99 |
| 9 | 6.63 | 9.12 | 32.38 | 6.29 |
| 10 | - | - | 2.65 | 6.99 |
| 11 | - | - | 2.34 | 7.17 |
| 12 | - | - | 7.30 | 7.00 |

**Table S6.** Mean height and adhesion for each area shown in Figure S7c and S8c for the data obtained with RTESPA-150 probe.

| Area | $MnPS_3@H_2O$ Thickness [nm] | $MnPS_3@H_2O$ Adhesion [nN] | $MnPS_3@PVP$ Thickness [nm] | $MnPS_3@PVP$ Adhesion [nN] |
|---|---|---|---|---|
| 1 | 3.38 | 1.65 | 5.55 | 0.93 |
| 2 | 1.71 | 1.51 | 13.59 | 0.89 |
| 3 | 1.75 | 1.54 | 6.73 | 0.96 |
| 4 | 2.09 | 1.42 | 2.71 | 1.01 |
| 5 | 1.98 | 1.47 | 5.04 | 1.09 |
| 6 | - | - | 2.38 | 1.12 |

**Table S7.** Mean height and adhesion for each area shown in Figure S9c and S10c for the data obtained with ScanAsyst Air probe.

| Area | $MnPS_3@H_2O$ Thickness [nm] | $MnPS_3@H_2O$ Adhesion [nN] | $MnPS_3@PVP$ Thickness [nm] | $MnPS_3@PVP$ Adhesion [nN] |
|---|---|---|---|---|
| 1 | 1.26 | 1.32 | 2.38 | 2.85 |
| 2 | 0.73 | 1.60 | 2.35 | 3.12 |
| 3 | 0.54 | 1.57 | 3.39 | 3.03 |
| 4 | 1.74 | 1.57 | 2.28 | 3.13 |
| 5 | 0.55 | 1.53 | 10.06 | 3.13 |
| 6 | 1.33 | 1.63 | 22.51 | 3.03 |
| 7 | 0.69 | 1.43 | 2.26 | 3.20 |
| 8 | 1.18 | 1.34 | 9.40 | 3.19 |
| 9 | - | - | 42.71 | 2.95 |

**5. Influence of the applied PeakForce Setpoint on the adhesion response of the probe.**

To evaluate the effect that the PFS can have on the measurements, we have compared the results yielded by SAA probes with the ones obtained with Tap300G probes. We have to consider the following:

**Tap 300G** (Stiffness: 40 N/m) the PFS applied has been kept at 25 nN

**SAA** (Stiffness: 0.4 N/m) the PFS applied has been kept at 1 nN

The PFS is always fixed to the minimum value needed to get stable and reliable data, so avoiding unnecessary damage of the tip or the sample during the measurements. In Figure S22, the indentation and height data are plotted for the same areas studied in the adhesion-height analysis in the main text. As can be observed, by keeping constant PFS values, the degree of indentation and the thickness of the layers under study cannot be correlated, moreover, the range of indentation variations is very narrow in all cases but even lower for the SAA probe (less stiff).

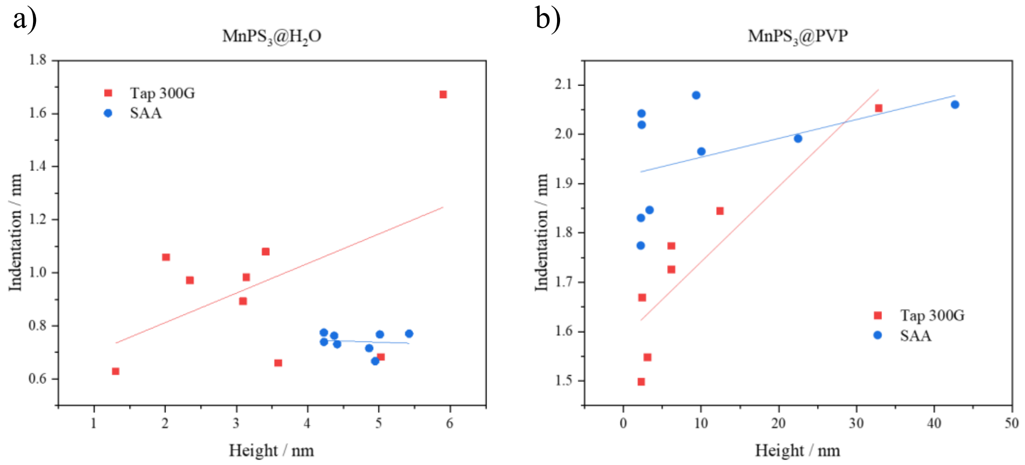


**Figure S22.** Indentation *vs* height values registered on each area studied in the main text with the probes SAA and Tap 300G: a) $MnPS_3$@$H_2O$ samples, and b) $MnPS_3$@PVP samples.

One step forward, we have performed additional adhesion measurements by using a SAA probe to scan 2 new samples, one with $MnPS_3@H_2O$ (Figure S23) and another with $MnPS_3@PVP$ (Figure S24). In these new measurements, a continuous increase of the applied force to the tip (PFS) was performed. In both samples, indentation values measured on $MnPS_3@H_2O$ and $MnPS_3@PVP$ flakes were almost invariable when low PFS were applied (PFS < 2), however, for larger PFS values, the indentation increased significantly (Figure S25a).

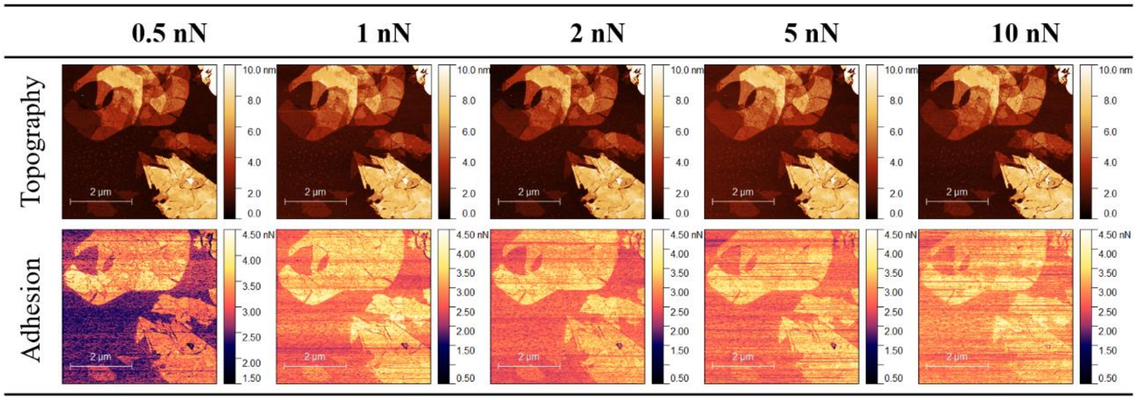


**Figure S23.** AFM images of $MnPS_3@H_2O$ obtained with PFS between 0.5nN and 10nN with a SAA probe. Top row: topography images; bottom row: adhesion signal.

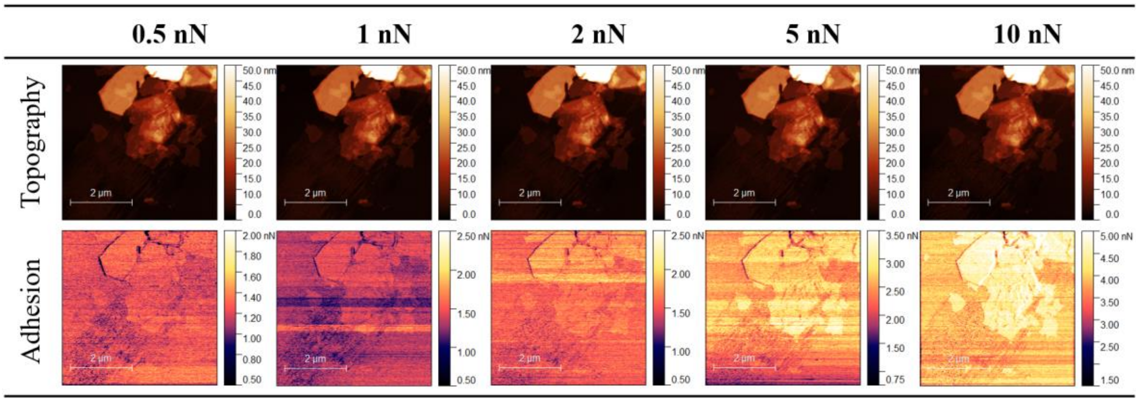


**Figure S24.** AFM images of $MnPS_3@PVP$ obtained with PFS between 0.5nN and 10nN with a SAA probe. Top row: topography images; bottom row: adhesion signal.

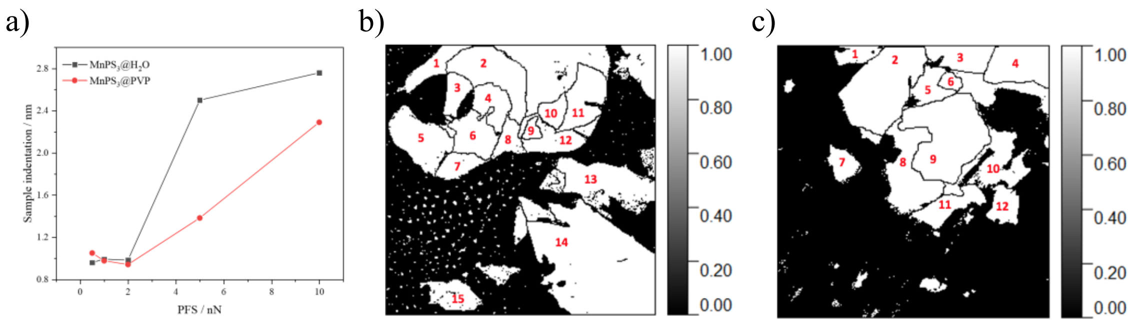


**Figure S25.** a) Mean Indentation values *vs* PFS applied measured with a SAA probe on $MnPS_3$@$H_2O$ and $MnPS_3$@PVP samples. b) Distribution of AFM image areas for further study on $MnPS_3$@$H_2O$ sample and c) Distribution of AFM image areas for further study on $MnPS_3$@PVP sample.

From figures S23 and S24, several areas were highlighted (Figure S25, b and c) and the adhesion was analyzed individually for each area on each image and PFS. The results obtained were plotted on Figure S26 as raw adhesion and RA. Regarding the adhesion, there is a general trend for both kind of samples: higher PFS induce broader dispersion in adhesion values. However, while for $MnPS_3$@$H_2O$ there is no relation between PFS, indentation, and raw adhesion or RA value, when $MnPS_3$@PVP sample is inspected, the adhesion absolute values increase with the increase of the applied force to the tip (PFS) but are completely independent of the 2Dm thickness. More interestingly, when RA values instead of absolute adhesion values are used, they result almost independent of both, PFS (and indentation) and layers thicknesses (highlighting once more the relevance of the use of RA for consistency when comparing different experiments). The $MnPS_3$@PVP dependencies can be attributed to the softer organic layer on the surface, which is more affected by a stronger contact, whereas $MnPS_3$@$H_2O$ does not exhibit such dependencies.

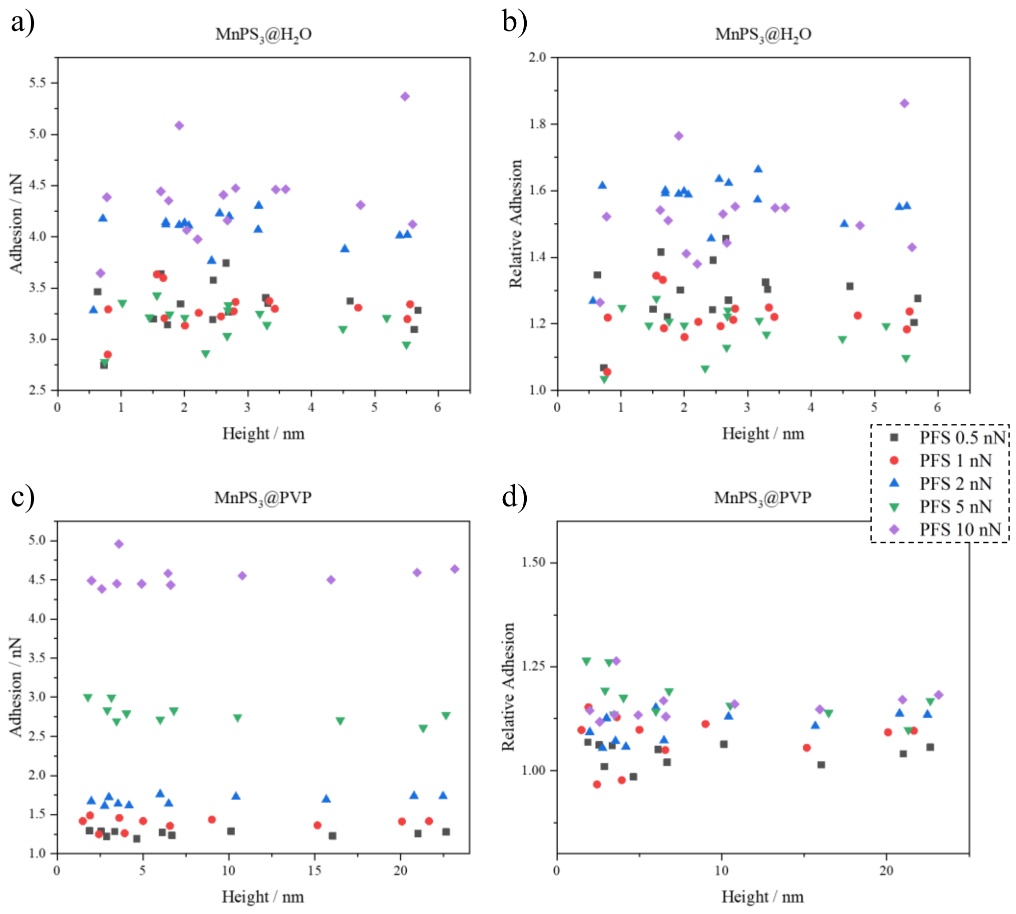


**Figure S26.** Top: Plots of $MnPS_3$@$H_2O$ samples adhesion (a) and relative adhesion (b) *vs* 2Dm thickness with a SAA probe. Bottom: Plots of $MnPS_3$@PVP samples adhesion (c) and relative adhesion (d) *vs* 2Dm thickness with a SAA probe.

## 6. Statistical analysis of RA for $MnPS_3$@$H_2O$ and $MnPS_3$@PVP samples.

For performing a statistical study of the results obtained with SAA and RTESPA probes, 2-3 images have been analysed on each probe-sample couple ensuring that we have enough data for the further ANOVA and Shapiro analysis.

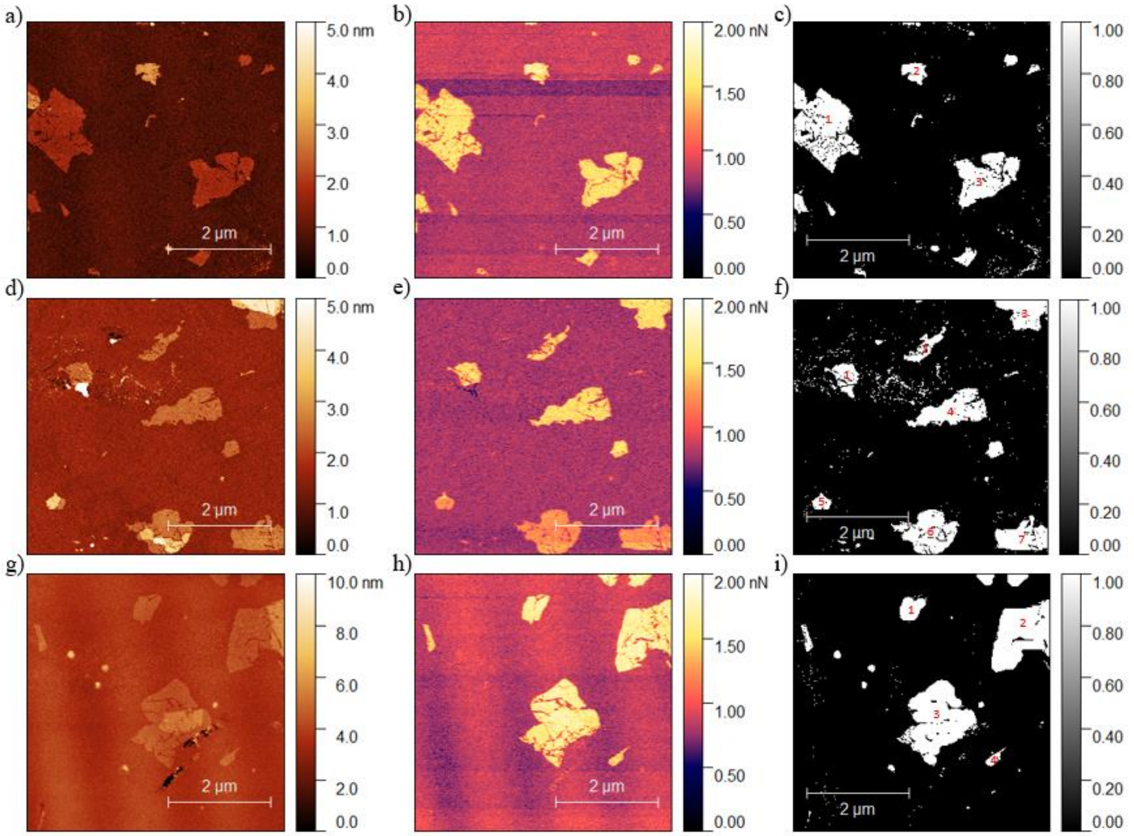


**Figure S27.** AFM images taken with ScanAsyst Air probe on $MnPS_3$@$H_2O$ samples. First sample: a) topography channel, b) adhesion signal and c) areas selected for further analysis. Second Sample: d) topography channel, e) adhesion signal and f) areas selected for further analysis. Third Sample: g) topography channel, h) adhesion signal and i) areas selected for further analysis.

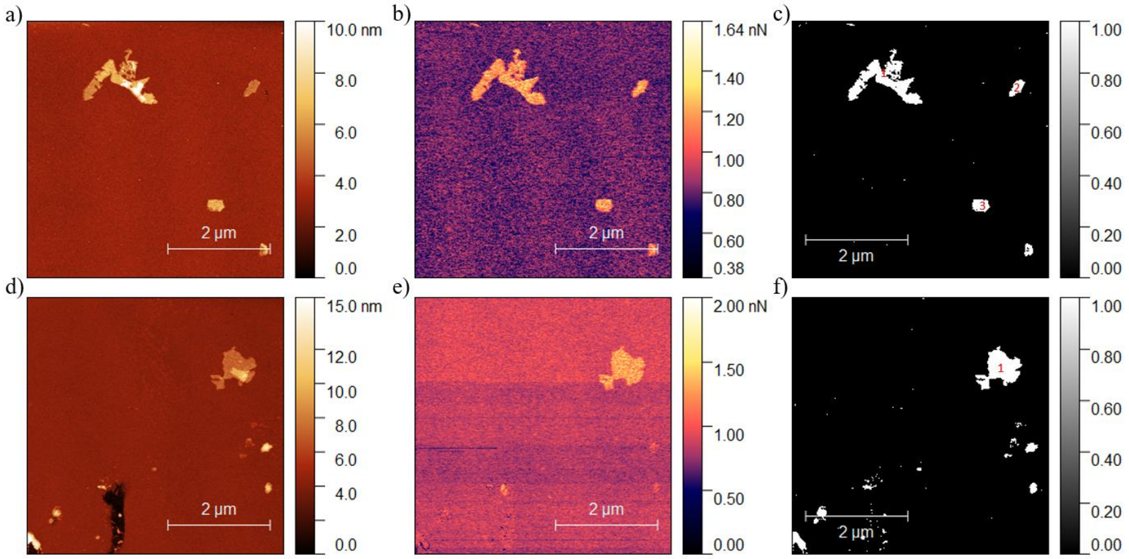


**Figure S28.** AFM images taken with ScanAsyst Air probe on $MnPS_3$@PVP samples. First sample: a) topography channel, b) adhesion signal and c) areas selected for further analysis. Second Sample: d) topography channel, e) adhesion signal and f) areas selected for further analysis.

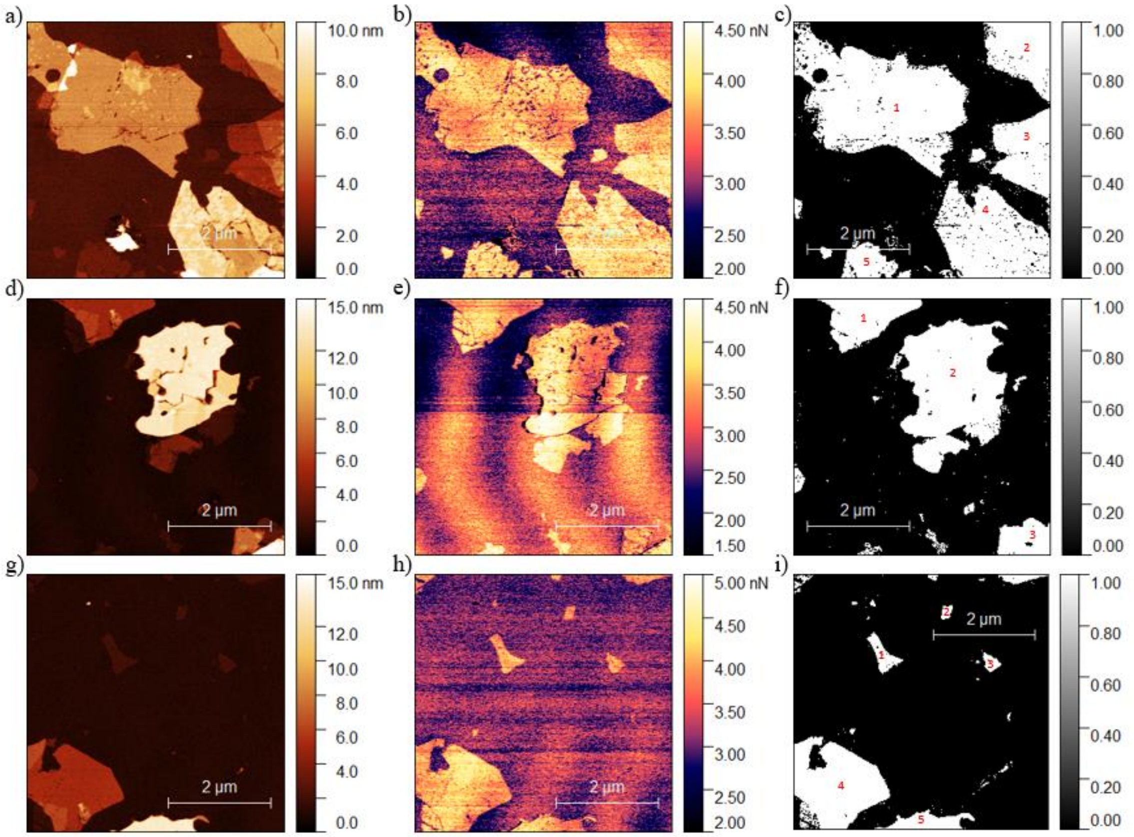


**Figure S29.** AFM images taken with RTESPA 150 probe on $MnPS_3@H_2O$ samples. First sample: a) topography channel, b) adhesion signal and c) areas selected for further analysis. Second Sample: d) topography channel, e) adhesion signal and f) areas selected for further analysis. Third Sample: g) topography channel, h) adhesion signal and i) areas selected for further analysis.

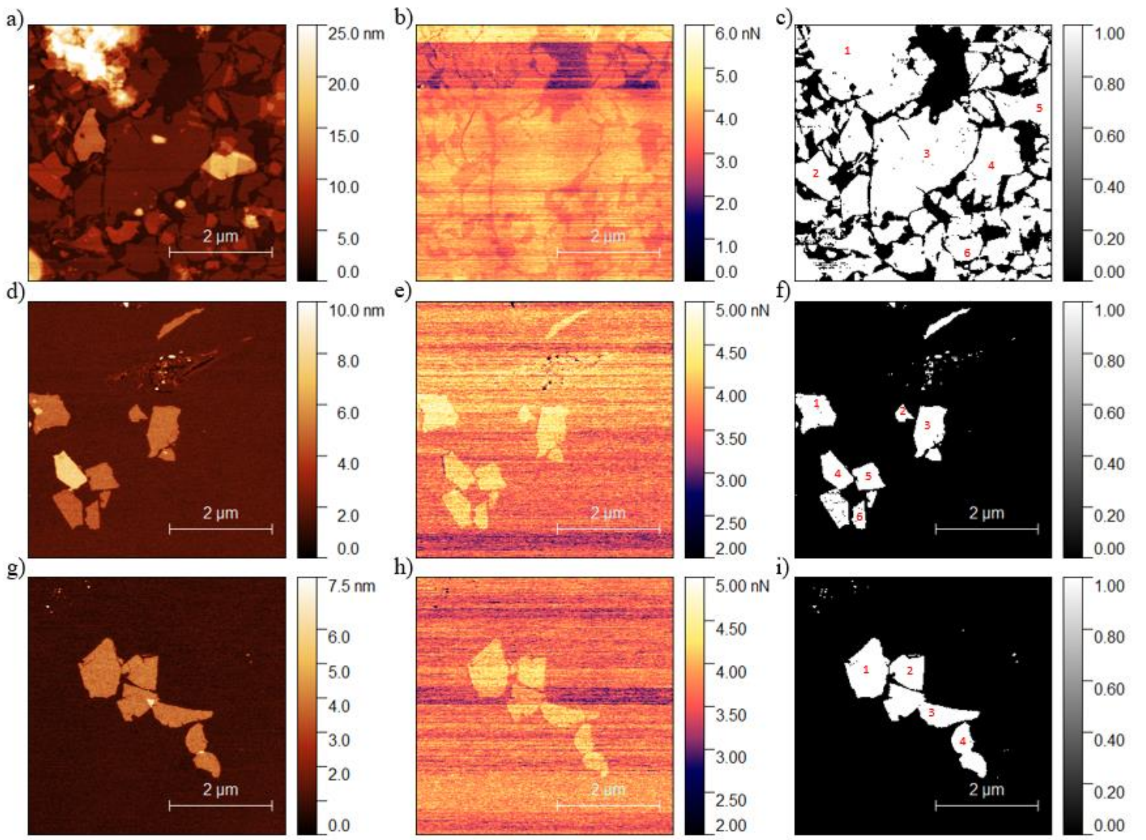


**Figure S30.** AFM images taken with RTESPA 150 probe on $MnPS_3$@PVP samples. First sample: a) topography channel, b) adhesion signal and c) areas selected for further analysis. Second Sample: d) topography channel, e) adhesion signal and f) areas selected for further analysis. Third Sample: g) topography channel, h) adhesion signal and i) areas selected for further analysis.

We have performed a typical ANOVA test (analysis of variance) to test if the groups of samples within each probe are different enough based on their RA values. ANOVA methods are a powerful tool usually employed to determine if there are significant differences between groups of data. It measures the differences between the means of the different groups. If the p-value is below a defined significance level (usually 0.05) means that there is enough evidence to reject the null hypothesis (the assumption that there are no differences between groups). By using Rstudio we compared with ANOVA (specifically through a t-test) the differences of the two types of samples ($MnPS_3$@PVP and $MnPS_3$@$H_2O$) for each probe independently (ScanAsyst Air and RTESPA 150).

First, we ran a Shapiro-Wilk normality test, which assesses whether a given sample follows a normal distribution. It specifically checks for normality. It retrieves a W metric, for which values close to 1 indicate that the data is close to a normal distribution, whereas values close to 0 suggest no normality. We have obtained values of W of 0.887 and 0.962 for data coming from ScanAsyst Air and RTESPA 150 experiments, respectively.

Being confirmed that our data follows normality, we can apply the well-known t-test to determine if there are significant differences between the two groups of samples. We have obtained values of p-value of $2.007 \cdot 10^{-6}$ and $7.219 \cdot 10^{-6}$ while comparing RA values with sample type for each of the two experiments. This means that for each probe, the data obtained for $MnPS_3$@PVP and $MnPS_3$@$H_2O$ are statistically significant.

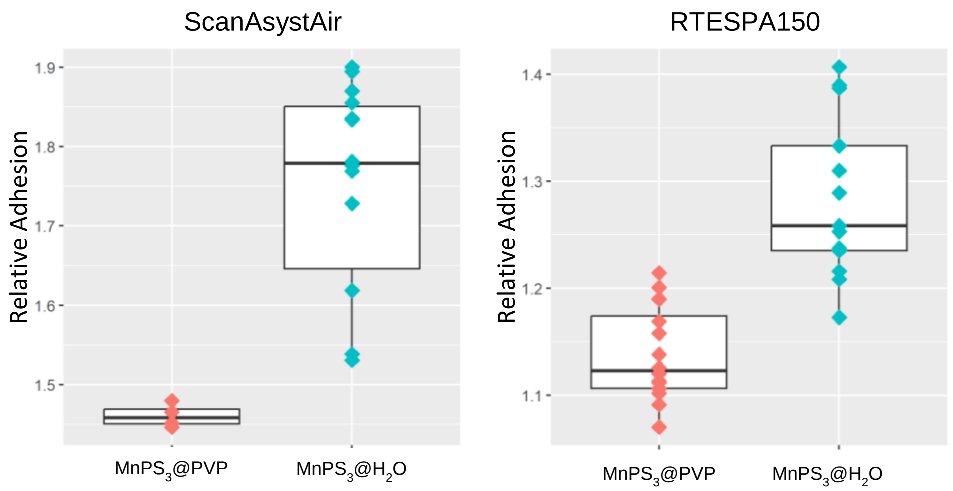


**Figure S31.** Boxplots of the obtained data in each of the experiments. The plot on the left corresponds to ScanAsyst Air probe, the plot on the right corresponds to RTESPA 150 probe classified in both cases by type of sample. It can be seen a slightly higher overlapping of the data in the second plot.

## 7. Classification process.

K-means is one of the most popular clustering methods which aims to partition *n* observations into *k* clusters in which each observation belongs to the cluster with the nearest mean. It is one of the simplest unsupervised learning algorithms that solves the well-known clustering problem [1]. This method could be summarized understanding the basic idea of the algorithm: relocate each point to its new nearest center (starting from an initial point not optimized), update the clustering centers by calculating the mean of the member points, and repeat the relocating-and-updating process until convergence criteria is satisfied [2].

The classification process was accomplished using Python through scikit-learn package. Following the last idea, two categories were chosen that correspond to the two types of materials on the sample: Water and PVP. Convergence criteria were defined by default. As one could observe, both problem samples are well-defined, and their centroids are distant. To verify the performance of the classification, it was suggested the use of two evaluation metrics: Silhouette and Inertia analysis.

Silhouette analysis helps to find the separation distance between the resulting clusters, its score has a range of [-1, 1]. Thus, if the Silhouette score has a value near +1 that indicates the sample is far away from the neighboring clusters. A value of 0 indicates that the sample is on or very close to the decision boundary between two neighboring clusters and negative values indicate that those samples might have been assigned to the wrong cluster [3]. On the other hand, Inertia tries to measure the compactness of each cluster by calculating the sum of squared distances of samples to their closest cluster center, weighted by the sample weights if provided. Lower inertia score indicates tighter and well-separated clusters.

**Table S8**: Obtained inertia and Silhouette scores validating the accuracy of k-means method while classifying data from mixed samples.

| Data | Inertia.score | Silhouette.score |
|---|---|---|
| SAA $MnPS_3$@Mix | 0.005230 | 0.936650 |
| Rtespa150 $MnPS_3$@Mix | 0.080268 | 0.716411 |

## 8. Comparison with mechanically exfoliated flakes.

As was commented briefly in the main text, the results obtained for $MnPS_3$@$H_2O$ and $MnPS_3$@PVP samples were compared to ME-$MnPS_3$ flakes. These results have been assessed with two different types of probes, ScanAsyst and RTESPA-150. The comparison of the data is discussed in the main text, and here it is possible to find all the AFM images that yielded the data. (Note that $MnPS_3$@$H_2O$ and $MnPS_3$@PVP samples were deeply studied in ESI section 6, hence, those data have been already shown in Figure S27-S30). In the next figures, the images obtained for ME-$MnPS_3$ samples with SAA and RTESPA-150 probes are presented.

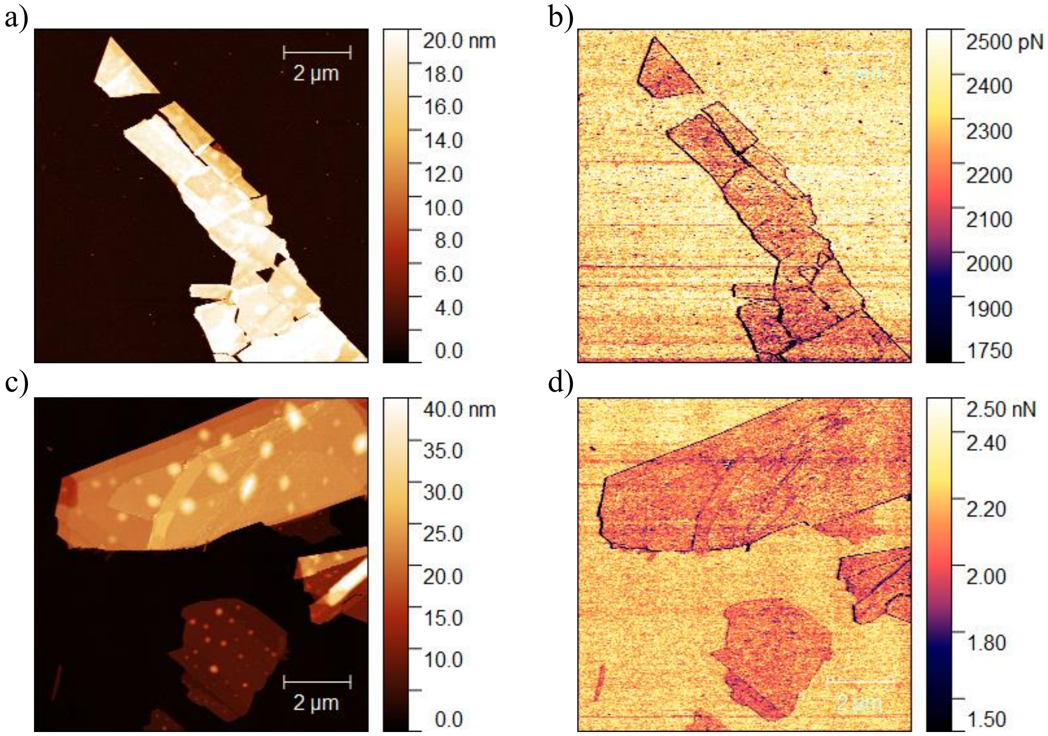


**Figure S32.** Topography a), c) and Adhesion b), d) images measured with SAA(3) on ME-$MnPS_3$ samples for collecting the data plotted in Figure 7.

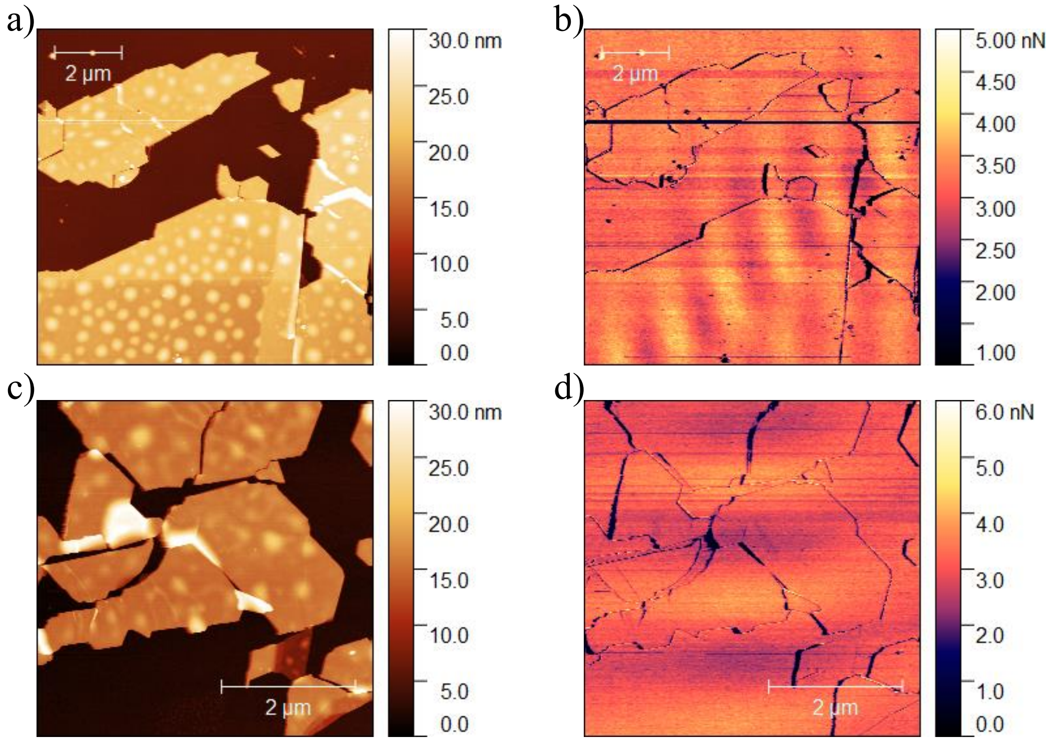


**Figure S33.** Topography a), c) and Adhesion b), d) images measured with RTESPA-150 on ME-$MnPS_3$ samples for collecting the data plotted in Figure 7.

## 9. Analysis of $MoS_2$ samples

$MoS_2$@$H_2O$ and $MoS_2$@PVP samples were prepared on Si/$SiO_2$ substrates, and the RA was studied taking several pictures on each sample. The analysis of these samples was done with a SAA probe. AFM pictures can be found in Figure S34-S35, and the mean RA values obtained from these pictures are represented in Figure S36.

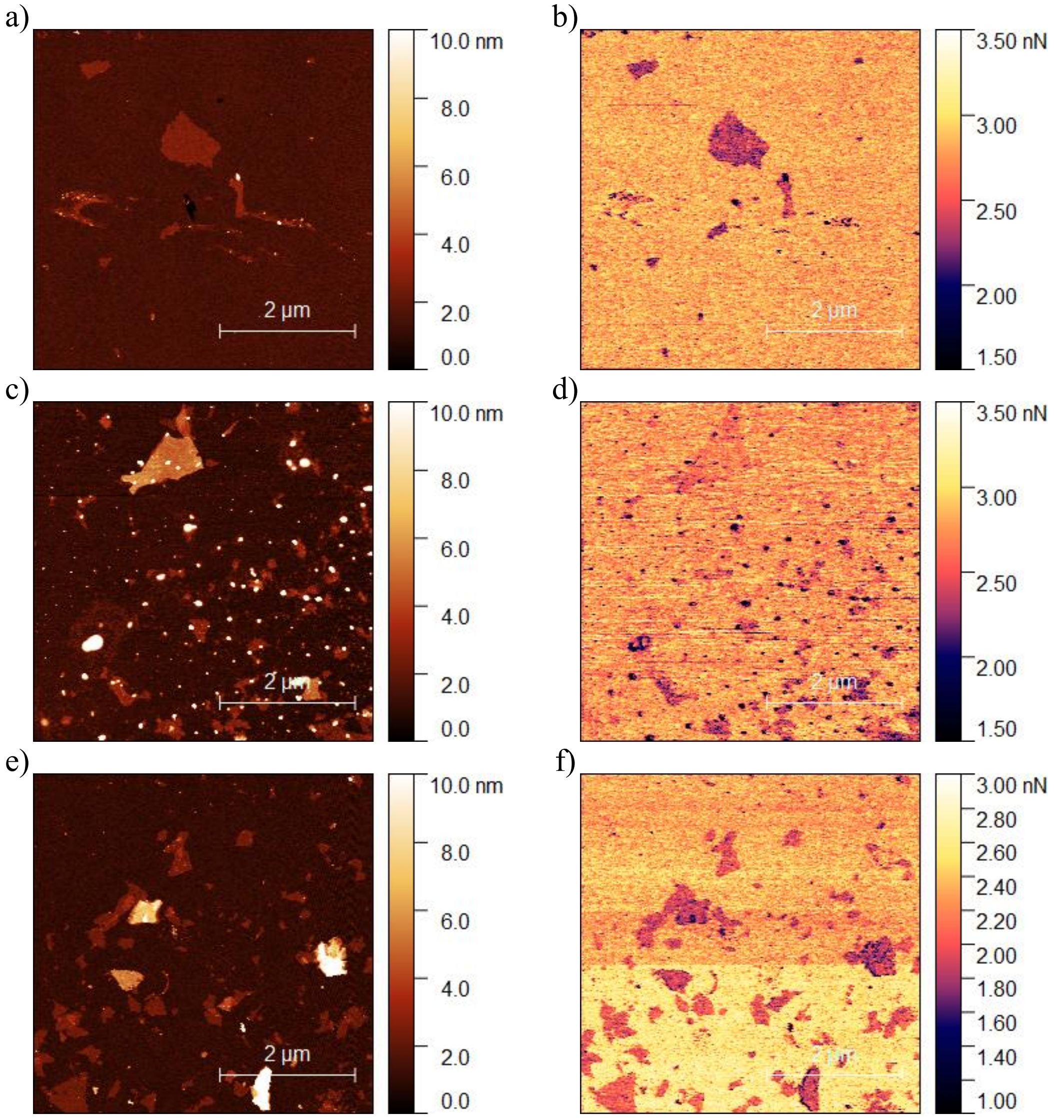


**Figure S34** Topography a), c), e) and Adhesion b), d), f) images measured with SAA on $MoS_2$@$H_2O$ samples for collecting the data plotted in Figure S36.

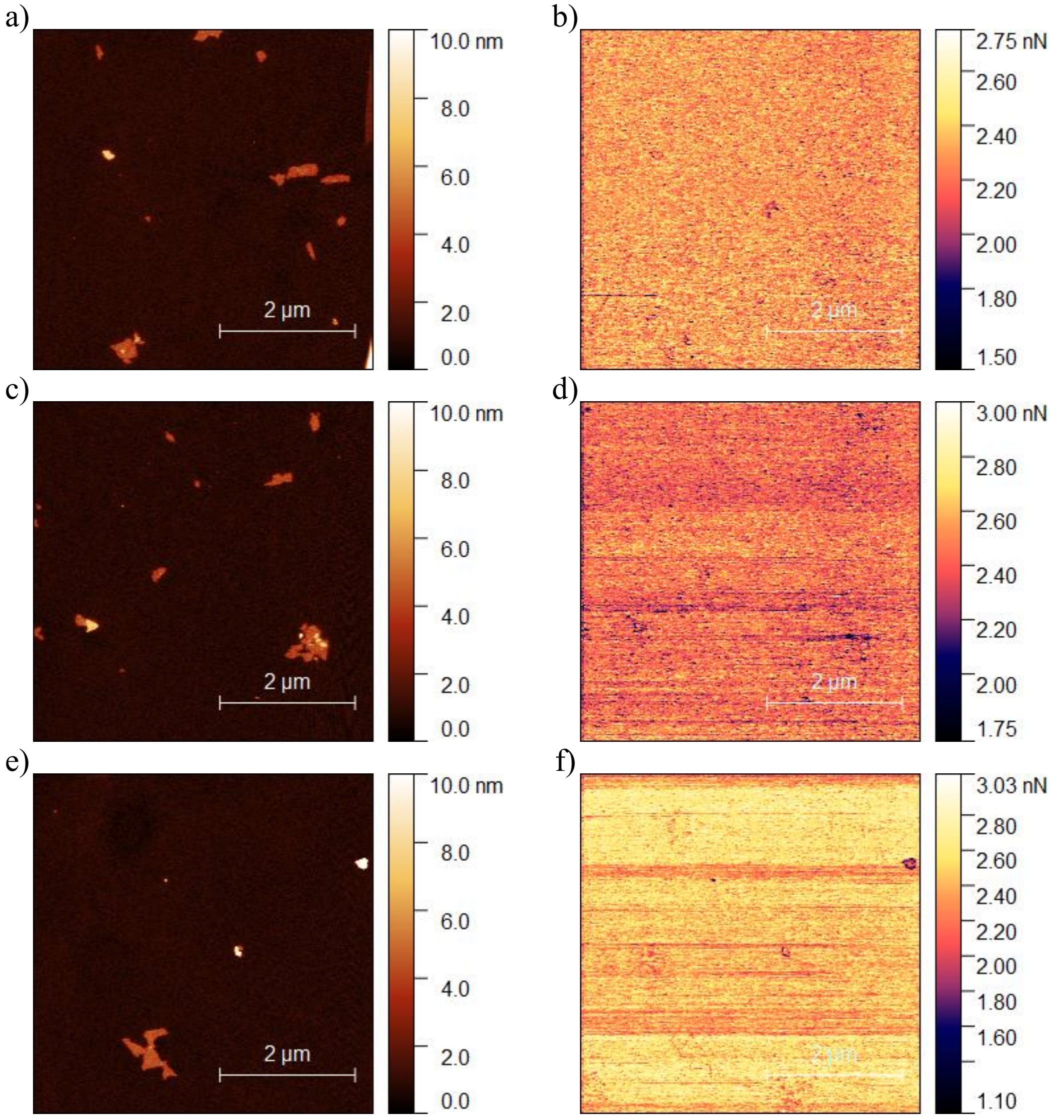


**Figure S35** Topography a), c), e) and Adhesion b), d), f) images measured with SAA on $MoS_2$@PVP samples for collecting the data plotted in Figure S36.

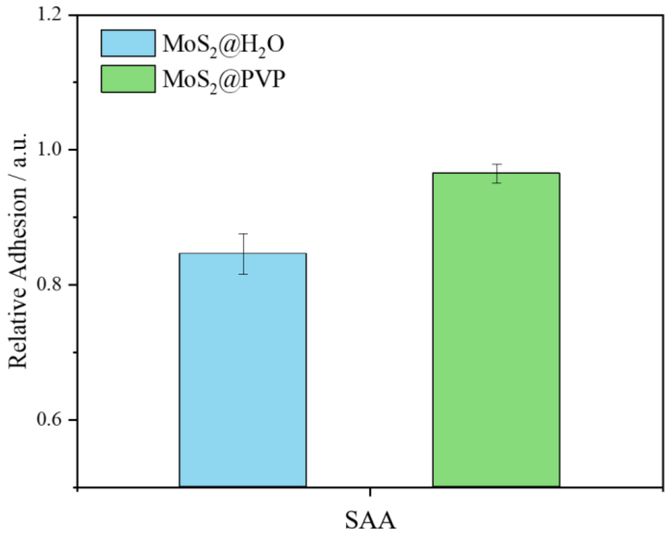


**Figure S36** RA values obtained on $MoS_2$@$H_2O$ flakes and on $MoS_2$@PVP with a SAA probe.

## 10. References

(1) Singh, K., Malik, D., & Sharma, N. Evolving limitations in K-means algorithm in data mining and their removal. International *Journal of Computational Engineering & Management*, **2011,** *12(1)*, 105-109.

(2) Sammut, C., & Webb, G. I. (Eds.). Encyclopedia of machine learning, Springer Science & Business Media, **2011.**

(3) Vysala, A., & Gomes, D. J. Evaluating and validating cluster results. arXiv preprint arXiv:2007.08034, **2020**.
